# Parkinson’s disease patients’ short chain fatty acids production capacity after in vitro fecal fiber fermentation

**DOI:** 10.1038/s41531-021-00215-5

**Published:** 2021-08-13

**Authors:** Florence Baert, Christophe Matthys, Jarissa Maselyne, Christof Van Poucke, Els Van Coillie, Bruno Bergmans, Geertrui Vlaemynck

**Affiliations:** 1 Department Technology and Food, Flanders Research Institute for Agriculture, Fisheries and Food, Melle, Belgium; 2 Clinical and Experimental Endocrinology, Department of Chronic Diseases and Metabolism, KU Leuven, O&N I, Leuven, Belgium; 3 Department of Endocrinology, University Hospitals Leuven, Campus Gasthuisberg, Leuven, Belgium; 4 Department of Neurology, AZ Sint-Jan Brugge-Oostende AV, Bruges, Belgium; 5 Department of Neurology, University Hospitals Ghent, Ghent, Belgium

**Keywords:** Parkinson's disease, Microbiology

## Abstract

Animal models indicate that butyrate might reduce motor symptoms in Parkinson’s disease. Some dietary fibers are butyrogenic, but in Parkinson’s disease patients their butyrate stimulating capacity is unknown. Therefore, we investigated different fiber supplements’ effects on short-chain fatty acid production, along with potential underlying mechanisms, in Parkinson’s patients and age-matched healthy controls. Finally, it was investigated if this butyrate production could be confirmed by using fiber-rich vegetables. Different fibers (*n* = 40) were evaluated by in vitro fermentation experiments with fecal samples of Parkinson’s patients (*n* = 24) and age-matched healthy volunteers (*n* = 39). Short-chain fatty acid production was analyzed by headspace solid-phase micro-extraction gas chromatography-mass spectrometry. *Clostridium coccoides* and *C. leptum* were quantified through 16S-rRNA gene-targeted group-specific qPCR. Factors influencing short-chain fatty acid production were investigated using linear mixed models. After fiber fermentation, butyrate concentration varied between 25.6 ± 16.5 µmol/g and 203.8 ± 91.9 µmol/g for Parkinson’s patients and between 52.7 ± 13.0 µmol/g and 229.5 ± 42.8 µmol/g for controls. Inulin had the largest effect, while xanthan gum had the lowest production. Similar to fiber supplements, inulin-rich vegetables, but also fungal β-glucans, stimulated butyrate production most of all vegetable fibers. Parkinson’s disease diagnosis limited short-chain fatty acid production and was negatively associated with butyrate producers. Butyrate kinetics during 48 h fermentation demonstrated a time lag effect in Parkinson’s patients, especially in fructo-oligosaccharide fermentation. Butyrate production can be stimulated in Parkinson’s patients, however, remains reduced compared to healthy controls. This is a first step in investigating dietary fiber’s potential to increase short-chain fatty acids in Parkinson’s disease.

## Introduction

As reviewed by Elfil et al. (2020), changes in the gut microbiome composition are thought to play a role in the pathophysiology of Parkinson’s disease (PD) and could be a potential target in future therapies^1^. The gut microbiome composition of PD patients is characterized by a lower number of butyrate-producing bacteria and a more pro-inflammatory profile^2–7^, combined with lower fecal short-chain fatty acids (SCFA) concentrations^5^. SCFA are hypothesized to be important gut-brain axis mediators^8^. In vitro studies have demonstrated that SCFA cross the blood-brain barrier, moreover low concentrations of butyrate and propionate have been reported in healthy volunteers’ brains^9,10^. PD animal models demonstrated that butyrate administration improves motor deficits, reduces inflammation and dopamine deficiency^11–13^. Matt et al. (2018) showed that both intra-peritoneal butyrate administration and a high dietary fiber diet resulted in reduced expression of pro-inflammatory genes in aged mice’s brains^14^. In contrast, results by Sampson et al. (2016) demonstrated that administration of an SCFA mixture promoted neuroinflammation in a germ-free PD mouse model^15^. However, the effect of the SCFA may depend on the concentration and on the composition of the SCFA mixture^16^. The administered concentration of the SCFA mixture may not resemble the concentration of microbial produced SCFA^16^. In fact, beneficial effects of administration of a low dose of butyrate in a mouse model of autism have been reported, whereas a high dose did not exert any effects^17^. Except for the study by Sampson et al. (2016), the above-mentioned animal studies suggest that SCFA, particularly butyrate, are noteworthy to investigate further. The hypothesis is that enhancing colonic SCFA production would be beneficial in PD.

Colonic SCFA concentrations can be increased by administering butyrate as such (both oral and rectal) and/or by increasing the butyrate-producing bacteria through fecal transplantation, probiotics, or dietary fiber^18–25^. Disadvantages of enema’s, fecal transplantation, or probiotics compared to fiber may be the patient’s discomfort and/or hesitance towards their use^26^. Furthermore, manufacturing and digestion can decrease probiotics’ viability, thereby limiting their effects^27^. Hence, dietary fiber consumption could be an acceptable option. Especially as PD patients have a higher daily fiber intake compared to healthy controls (HC)^28–30^.

Following the suggestion of Elfil et al. (2020) of SCFA modification as a potential therapeutic strategy^1^, we hypothesize that a high fiber diet can increase butyrate production in PD patients through colonic fiber fermentation. Although a first step is to evaluate the SCFA producing capacity of PD patients’ gut microbiota.

Therefore, we aim to increase SCFA production in fecal samples of PD patients through in vitro fermentation experiments with different types of fiber supplements, compared to SCFA production in fecal samples of age-matched HC. By investigating fibers’ effects on butyrate-producing bacteria and SCFA production kinetics, we aim to increase understanding of mechanisms behind colonic SCFA production in PD patients. Finally, we evaluate whether vegetable and quinoa fiber can confirm the in vitro SCFA production results obtained by fiber supplements.

## Results

In total, 63 participants were included, 24 PD patients and 39 HC. A minimum of 4 fecal samples of both PD patients and HC were used per fiber substrate, see also Supplementary Table 1.

The mean Hoehn and Yahr score of PD patients was 2.3 ± 0.5, ranging from 1.5–3. An overview of all participants’ characteristics is shown in Table 1. A significant difference in the ratio of men to women (*p* = 0.00007), BMI (*p* = 0.01), reported weight loss (*p* = 0.02), and antidepressant intake (*p* = 0.006) was found between PD patients and HC.

**Table 1:** Overview of participant’s characteristics.

	PD patients (*n* = 24)	Healthy volunteers (*n* = 39)	*P-*value
***Sex***			
Men/women	21/3	14/25	0.00007
***Age***			
Age (years), mean ± SD	62.5 ± 6.9	60.5 ± 4.4	0.13
Duration of disease (years), mean ± SD	6.1 ± 5.0	–	–
Duration of disease (years), minimum–maximum	1–18	–	–
BMI (kg/m²), mean ± SD	24.2 ± 2.0	22.9 ± 1.9	0.01
Dietary fiber intake (g/day), mean ± SD	22.9 ± 9.7	24.5 ± 10.5	0.54
Bristol Stool Form Scale			0.11
Type 1 (%)	8.3	2.6	
Type 2 (%)	12.5	10.3	
Type 3 (%)	37.5	12.8	
Type 4 (%)	33.3	61.5	
Type 5 (%)	8.3	10.3	
Type 6 (%)	0.0	2.6	
Type 7 (%)	0.0	0.0	
Hoehn and Yahr score, mean ± SD (*n* = 18)	2.3 ± 0.5	–	–
Hoehn and Yahr score, minimum–maximum	1.5-3.0	–	–
Weight loss reported by participants (%)	29.2	5.7	0.02
Weight loss reported by participants (kg), mean ± SD	10.5 ± 7.9	2.8 ± 0.4	
Weight loss reported by participants (kg), minimum–maximum	3–20	2.5–3	
***Medication intake***			
Antidepressants (%)	20.8	0.0	0.006
Medication for high blood pressure (%)	20.8	15.4	0.73
Medication for high cholesterol levels (%)	20.8	20.5	1.00
Anti-inflammatory drugs (%)	4.2	5.1	1.00
Parkinson’s medication (%)	91.7	–	–
Levodopa + DOPA decarboxylase inhibitor (%)	75.0	–	–
Catechol-O-methyltransferase (COMT) inhibitor (%)	29.2	–	–
Dopamine agonists (%)	45.8	–	–
Monoamine oxidase-B (MAO B) inhibitor (%)	62.5	–	–
Amantadine (%)	4.2	–	–
Anticholinergics (%)	8.3	–	–
***Predominant Parkinson’s disease-related complaints***			
Movement issues (%)	83.3	–	–
Bowel problems (%)	20.8	–	–
Negative mood (%)	4.2	–	–
Speech difficulties (%)	4.2	–	–
Difficulties with eating/drinking (%)	8.3	–	–
Hypersalivation (%)	8.3	–	–

*SD* standard deviation, Bristol Stool Form Scale (BSFS) type 1 indicates separate hard to pass fecal lumps, BSFS type 2 indicates lumpy, sausage-shaped feces, BSFS type 3 indicates sausage-shaped feces with cracks on the surface, BSFS type 4 indicates smooth, sausage-shaped feces, BSFS type 5 indicates soft fecal blobs with sharp edges (easy to pass), BSFS type 6 indicates mushy soft stools and BSFS type 7 indicates entirely liquid stools. Possible differences in sex, BSFS, and medication intake between PD patients and HC were analyzed using Fisher’s exact test. To evaluate potential differences in BMI, fiber intake, and age between PD and HC, Students *T*-test and Mann–Whitney U-test were used, depending on normality (evaluated by the Shapiro Wilk test).

### Fiber supplements

**Fig. 1:**
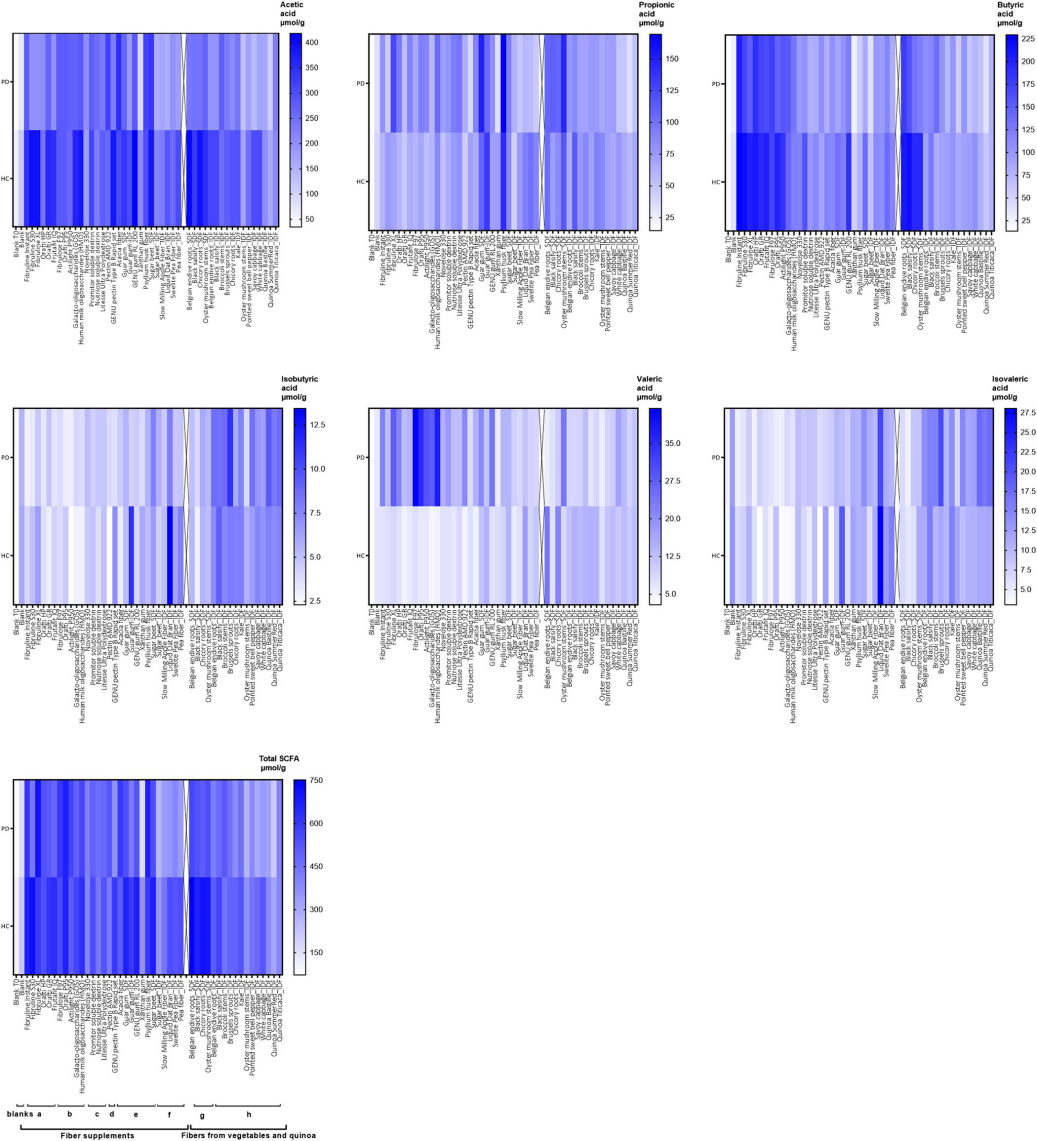
Overview of mean SCFA production per fiber in Parkinson’s Disease patients and healthy controls. **a** inulins; (**b**) oligosaccharides; (**c**) resistant starch/dextrin, polydextrose; (**d**) pectins; (**e**) gums; (**f**) hemicelluloses, cellulose, lignin; (**g**) vegetable soluble dietary fiber; (**h**) vegetable/quinoa insoluble dietary fiber; *SDF* soluble dietary fiber, *IDF* insoluble dietary fiber, *PD* Parkinson’s patients, *HC* healthy controls. Potential effects of factors on SCFA production were analyzed using linear mixed models, sex, fiber type, and PD diagnosis were added as fixed factors, the participant was added as a random factor. PD diagnosis resulted in an overall reduction of acetic acid (*p* = 0.002), butyric acid (*p* = 0.0001), and total SCFA (*p* = 0.004), compared to HC for the fiber supplement experiments (**a**–**f**). Similar effects of PD diagnosis on acetic acid (*p* = 0.01), butyric acid (*p* = 0.097), and total SCFA (*p* = 0.08) were observed in the vegetable/quinoa fiber experiments (**g**–**h**). Fibers significantly (*p* < 2.0E−16) influenced the production of all SCFA in both experiments.

Univariate analyses of baseline SCFA concentrations prior to fermentation (Blank T0) demonstrated that PD patients had significantly lower concentrations of acetate (*p* = 0.002) and total SCFA (*p* = 0.008) compared to HC, for butyrate a trend (*p* = 0.09) towards a lower concentration was found. Univariate analyses demonstrated that PD patients had overall significantly reduced acetate (*p* = 0.03) and butyrate (*p* = 0.01) production compared to HC, for total SCFA a trend (*p* = 0.09) towards lower production was found. Fiber supplements, except for xanthan gum, all stimulated butyrate production in PD and HC (see Fig. 1), however large inter-individual variability in SCFA production was found (see Supplementary Figs. 1–7). An overview of univariate analyses in all participants is given in Supplementary Table 2. PD medication, disease duration, and antidepressant intake’s influence on SCFA production was analyzed within PD patients. Levodopa was negatively associated with isobutyrate production (*p* = 0.04) and a trend towards lower isovalerate production (*p* = 0.05) was found. Catechol-O-methyltransferase (COMT) inhibitor was positively associated with total SCFA production (*p* = 0.04). Disease duration was positively associated with valerate production (*p* = 0.002).

**Fig. 2:**
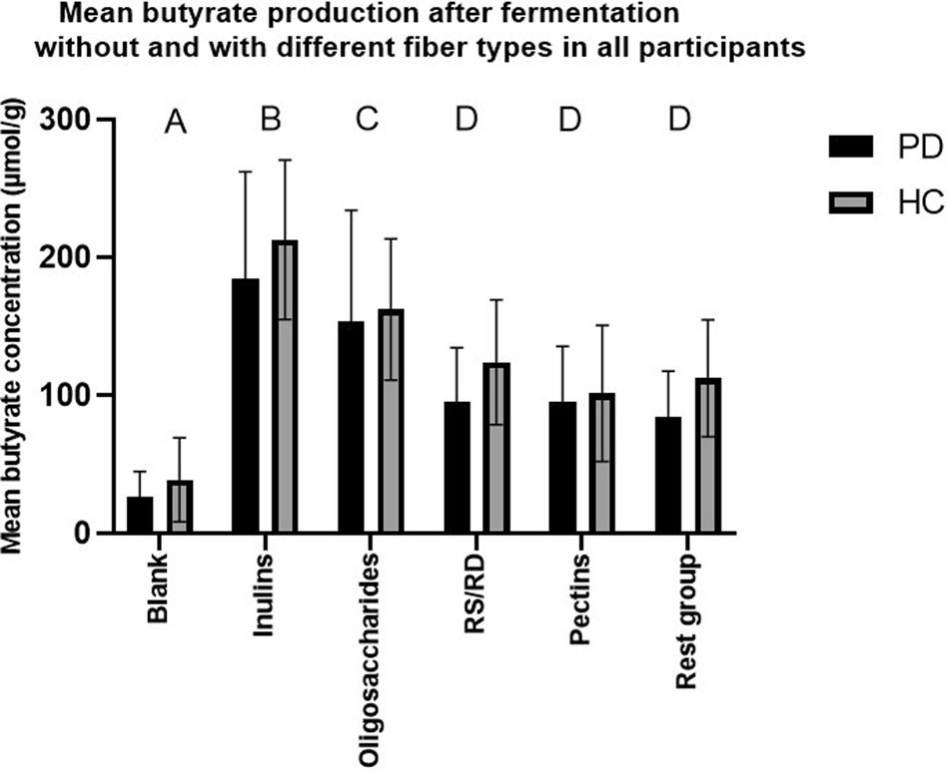
Mean butyrate concentration after 24h fermentation with different types of fiber supplements. Results presented as mean ± SD; different letters indicate significant differences in butyrate production between fiber types; RS resistant starch, RD resistant dextrin, oligosaccharides are fructo-, galacto- and human milk oligosaccharides; rest group comprises fibers consisting of hemicelluloses, cellulose, and lignin. Differences between fiber types were post-hoc analyzed by pairwise comparisons, using Tukey to correct for multiple testing. All significant differences observed, had a *p*-value < 0.0001.

Multivariate analysis demonstrated that fiber type and PD diagnosis were the strongest predictors of SCFA production, compared to other fixed factors. Fiber stimulated acetate, butyrate, and total SCFA production significantly, whereas PD diagnosis significantly limited their overall production. The final multivariate models are shown in Table 2A. Of all fiber types, inulins stimulated butyrate production most (*p* < 0.0001), see Figs 1 and 2. Oligosaccharides (*p* < 0.0001) increased butyrate production more compared to RS, pectins, and fibers consisting of hemicelluloses, cellulose, and lignin, see Fig. 2. Mean butyrate production increase in PD and HC per fiber type is shown in Supplementary Table 3 and Supplementary Fig. 8.

**Table 2:** Overview of linear mixed model multivariable analyses of fermentation experiments of fiber supplements (A), vegetable and quinoa fibers (B), kinetics (C1-7) and analyses of butyrate-producing bacteria (D).

	Acetic acid			Propionic acid			Butyric acid			Isobutyric acid			Valeric acid			Isovaleric acid			Total SCFA		
	Estimate (SD)	*P*-value	LSM	Estimate (SD)	*P*-value	LSM	Estimate (SD)	*P*-value	LSM	Estimate (SD)	*P*-value	LSM	Estimate (SD)	*P*-value	LSM	Estimate (SD)	*P*-value	LSM	Estimate (SD)	P -value	LSM
**A. SCFA~ Fiber type + PD diagnosis + Sex + (1|ID)**																					
Intercept	10.4 (0.5)	< 2.0E−16		3.5 (0.09)	< 2.0E−16		6 (0.4)	< 2.0E−16		2.4 (0.1)	< 2.0E−16		1.6 (0.16)	3.2E−14		2.4 (0.1)	< 2.0E−16		223.6 (25.5)	3.6E−12	
Fiber type		< 2.2E−16			< 2.2E−16			< 2.2E−16			< 2.0E−16			< 2.0E−16			< 2.0E−16			< 2.0E−16	
Blank	Ref.		90.4	Ref.		34	Ref.		28.9	Ref.		5.3	Ref.		5.5	Ref.		11.6	Ref.		197
Blank T0	−3.0 (0.3)		42.6	−0.8 (0.05)		14.8	−1.8 (0.2)		12.8	−1.0 (0.05)		1.9	−0.9 (0.07)		2.2	−1.2 (0.05)		3.4	−105.1 (13.9)		91.7
Inulins	7.2 (0.2)		279.2	0.7 (0.04)		66.6	8.3 (0.2)		187	−0.3 (0.04)		4	0.2 (0.05)		7	−0.8 (0.04)		5.4	395.1 (10.4)		592
Gums	7.2 (0.3)		278.6	1 (0.04)		88.7	4 (0.2)		87.5	−0.07 (0.04)		5	0.4 (0.06)		7.8	−0.4 (0.04)		8,1	338.7 (11.5)		536
Oligosaccharides	7.7 (0.3)		296.1	0.6 (0.04)		64.7	6.8 (0.2)		148	−0.5 (0.04)		3.2	0.1 (0.06)		6.2	−1.0 (0.04)		4.5	381 (11.7)		578
Pectins	7.7 (0.3)		296.6	0.5 (0.05)		54.1	4.3 (0.3)		94	−0.4 (0.05)		3.6	0.01 (0.08)		5.6	−0.8 (0.06)		5.5	300.3 (15)		497
Polydextrose	6 (0.5)		239.8	0.8 (0.07)		77.2	4.2 (0.4)		91.2	−0.2 (0.07)		4.7	0.2 (0.11)		6.5	−0.4 (0.08)		7.9	246.8 (20.9)		444
RD/RS	5.3 (0.3)		219.0	0.6 (0.05)		63.7	4.7 (0.2)		101.5	−0.2 (0.05)		4,4	0.3 (0.07)		7.4	−0.4 (0.05)		7,7	239.4 (13.1)		436.2
Rest group	6.4 (0.3)		251.6	0.7 (0.04)		70.6	4.3 (0.2)		92.8	0.2 (0.04)		6.2	0.6 (0.06)		9.7	−0.03 (0.04)		11.3	286.8 (11.9)		484
PD diagnosis		0.002			0.72			6.70E−05			0.046			0.68			0.4			0.004	
Healthy volunteers	Ref.		246.4	Ref.		52.2	Ref.		108	Ref.		4.8	Ref.		6.3	Ref.		7.3	Ref.		489
Parkinson’s patients	−2.5 (0.8)		174.1	−0.05 (0.14)		55	−2.3 (0.5)		65	−0.3 (0.1)		3.6	−0.1 (0.2)		5.7	−0.2 (0.2)		6.2	−122.2 (39.8)		367
Sex		0.3			0.5			0.04			0.14			0.14			0.31			0.08	
Female	Ref.		197,4	Ref.		51.1	Ref.		75.3	Ref.		3.8	Ref.		5	Ref.		6,1	Ref.		394
Male	0.8 (0.8)		220.2	0.09 (0.14)		56.2	1.1 (0.5)		95.8	0.2 (0.1)		4.6	0.4		7.2	0.2 (0.2)		7.4	68.5 (38.7)		463

**B. SCFA~ Fiber type + PD diagnosis + Sex + (1|ID)**																					
Intercept	10.2 (0.8)	2.2E−11		3.7 (0.15)	< 2.0E−16		5.9 (0.7)	2.90E−08		2.4 (0.2)	6.3E−13		1.8 (0.2)	7.30E−08		2.5 (0.2)	1.7E−13		233.2 (37.5)	2.9E−06	
Fiber type		< 2.0E−16			< 2.0E−16			< 2.0E−16			< 2.0E−16			< 2.0E−16			< 2.0E−16			< 2.0E−16	
Blank	Ref.		83.2	Ref.		35,4	Ref.		29.1	Ref.		5.9	Ref.		6.4	Ref.		11.9	Ref.		189
Blank T0	−2.9 (0.4)		38.5	−0.8 (0.04)		15.2	−1.8 (0.2)		13	−0.9 (0.06)		2.3	−0.9 (0.06)		2.6	−1.1 (0.05)		4	−102.03 (15)		86.6
IDF	4.9 (0.3)		197.8	0.7 (0.03)		69.2	3.6 (0.2)		80.3	0.2 (0.04)		7	0.4 (0.05)		9.3	−0.03 (0.04)		11.6	213 (11.7)		402
SDF	7.9 (0.3)		288.1	1 (0.04)		94.9	7.3 (0.2)		162	−0.3 (0.05)		4.6	0.2 (0.06)		8.2	−0.6 (0.05)		6.4	416 (13)		605
PD diagnosis		0.013			0.73			0.097			0.5			0.71			0.27			0.075	
Healthy volunteers	Ref.		176.6	Ref.		41.7	Ref.		73.4	Ref.		4.4	Ref.		5.6	Ref.		6.8	Ref.		373
Parkinson’s patients	−3.4 (1.2)		97.9	0.08 (0.22)		45.1	−1.8 (1.04)		45.9	0.2 (0.2)		5.1	0.1 (0.3)		6.4	0.3 (0.2)		8.8	−105.1 (55.8)		268
Sex		0.34			0.07			0.47			0.81			0.81			0.32			0.79	
Female	Ref.		120,2	Ref.		54,0	Ref.		52,9	Ref.		4,9	Ref.		6,2	Ref.		8,7	Ref.		312.3
Male	1.3 (1.3)		149.3	−0.4 (0.23)		34.8	0.8 (1.07)		65.1	−0.1 (0.2)		4.6	−0.08 (0.4)		5.7	−0.2 (0.2)		6.9	15.9 (57.7)		328

C1. SCFA~ Fiber type * PD diagnosis + Sex + (1|ID)																					
Intercept	14.6 (1.2)	< 0.0001		4.1 (0.5)	< 2.0E−16		13.3 (1.9)	< 0.0001		1.1 (0.5)	0.04		0.3 (0.8)	0.65		0.9 (0.7)	0.20		478.2 (53.6)	< 2.2E−16	
Fiber type		< 2.2E−16			0.02			< 2.2E−16			0.0006			0.0002			3.60E−08			< 2.2E−16	
Oligosaccharides	Ref.		182.9	Ref.		49.6	Ref.		101.0	Ref.		1.6	Ref.		1.7	Ref.		2.3	Ref.		374.3
Blank	−6.8 (0.7)		57.7	−0.2 (0.1)		30.8	−6.9 (0.4)		28.7	0.2 (0.1)		2.3	0.2 (0.1)		2.0	0.5 (0.1)		4.2	−309.6 (22.7)		136.3
RS	−4.4 (0.7)		101.6	−0.2 (0.1)		38,4	−4.1 (0.4)		56.5	0.05 (0.1)		2.1	0.4 (0.1)		2.4	0.4 (0.1)		3.9	−214.9 (22.7)		220.5
Inulins	−3.5 (0.7)		116.0	0.1 (0.1)		46.5	−1.6 (0.4)		84.8	0.3 (0.1)		2.1	0.2 (0.1)		1.9	0.2 (0.1)		2.8	−135.9 (22.7)		288.0
PD diagnosis		0.003			0.42			0.003			1.00			0.78			0.68			6.00E−04	
Healthy volunteers	Ref.		138.0	Ref.		38.0	Ref.		96.2	Ref.		2.0	Ref.		1.9	Ref.		3.5	Ref.		304.1
Parkinson’s patients	−3.8 (1.3)		85.3	0.4 (0.5)		43.4	−5.8 (1.9)		39.2	0.002 (0.5)		2.0	0.2 (0.8)		2.1	−0.3 (0.7)		2.9	−189.9 (55.3)		205.4
Sex		0.19			0.12			0.72			0.63			0.89			0.84			0.74	
Female	Ref.		93.9	Ref.		59.9	Ref.		70.5	Ref.		1.6	Ref.		1.8	Ref.		2.9	Ref.		263.7
Male	1.6 (1.2)		127.6	−0.8 (0.5)		27.5	−0.7 (2.0)		58.9	0.3 (0.6)		2.4	0.1 (0.8)		2.1	0.2 (0.8)		3.5	−17.9 (54.9)		245.8
Fiber type * PD diagnosis		0.15			0.03			1.50E−13			3.00E−04			0.46			0.04			3.50E−05	
Blank * PD diagnosis	1.7 (0.9)			−0.5 (0.2)			4.5 (0.6)			−0.02 (0.1)			−0.1 (0.1)			0.2 (0.1)			143.2 (32.1)		
RS * PD diagnosis	2 (0.9)			−0.2 (0.2)			3.1 (0.6)			0.3 (0.1)			−0.2 (0.1)			0.3 (0.1)			122.3 (32.6)		
Inulins * PD diagnosis	1.5 (0.9)			−0.4 (0.2)			1.6 (0.6)			−0.2 (0.1)			−0.2 (0.1)			0.007 (0.1)			99.3 (32.1)		

C2. SCFA~ Fiber type * PD diagnosis + Sex + (1|ID)																					
Intercept	15 (1.4)	< 0.0001		4.2 (0.4)	< 2.0E−16		14.5 (2.1)	< 0.0001		1.1 (0.5)	0.04		0.3 (0.8)	0.72		0.8 (0.7)	0.23		543.2 (60.2)	< 2.0E−16	
Fiber type		< 2.0E−16			2.00E−04			< 2.2E−16			1.40E−07			3.20E−05			6.40E−13			< 2.0E−16	
Oligosaccharides	Ref.		234.6	Ref.		60.5	Ref.		140	Ref.		1.6	Ref.		1.7	Ref.		2.2	Ref.		479
Blank	−7.3 (0.6)		62.9	−0.2 (0.1)		36.0	−8.1 (0.5)		32.1	0.4 (0.1)		2.8	0.4 (0.1)		2.6	0.8 (0.1)	< 0.0001	5.5	−364.3 (25.2)		156
RS	−4.4 (0.6)		113.3	−0.2 (0.1)		45.4	−4.8 (0.5)		72.0	0.1 (0.1)		2.1	0.5 (0.1)		2.6	0.5 (0.1)	0.0001	4.0	−250.3 (25.2)		262
Inulins	−3.6 (0.6)		141.9	0.2 (0.1)		61.3	−1.2 (0.5)		128	0.2 (0.1)		2.1	0.4 (0.1)		2.1	0.2 (0.1)	0.17	2.7	−135.2 (25.2)		370
PD diagnosis		0.29			0.11			0.02			0.91			0.690			0.72			0.06	
Healthy volunteers	Ref.		149.8	Ref.		42.6	Ref.		117	Ref.		2.1	Ref.		2.0	Ref.		3.6	Ref.		350
Parkinson’s patients	−1.5 (1.4)		113.6	0.6 (0.4)		57.8	−5.0 (2.1)		61.9	0.1 (0.5)		2.3	0.3 (0.8)		2.4	−0.2 (0.7)		3.2	−117.2 (62.6)		284
Sex		0.12			0.04			0.91			0.59			0.85			0.79			0.84	
Female	Ref.		107.6	Ref.		74.9	Ref.		89.5	Ref.		1.7	Ref.		2.0	Ref.		3.1	Ref.		311
Male	2.2 (1.4)		156.9	−0.8 (0.4)		32.9	−0.3 (2.2)		84.7	0.3 (0.6)		2.6	0.2		2.4	0.2 (0.7)		3.7	−12.3 (62.2)		323
Fiber type * PD diagnosis		0.63			0.003			1.70E-06			0.01			0.16			0.39			0.1	
Blank * PD diagnosis	−0.2 (0.8)			−0.6 (0.2)			3.8 (0.8)			0.002 (0.1)			−0.03 (0.2)			0.2 (0.2)			84.4 (35.7)		
RS * PD diagnosis	−0.6 (0,8)			−0.2 (0.2)			3 (0.8)			0.2 (0.1)			−0.2 (0.2)			0.2 (0.2)			67.4 (35.7)		
Inulins * PD diagnosis	0.5 (0.8)			−0.5 (0.2)			1.5 (0.8)			−0.2 (0.1)			−0.3 (0.2)			0.05 (0.2)			53.3 (35.7)		

C3. SCFA~ Fiber type * PD diagnosis + Sex + (1|ID)																					
Intercept	14.6 (1.0	<0.0001		4.4 (0.4)	< 2.2E**−**16		14 (1.7)	< 0.0001		1.2 (0.6)	0.06		0.4 (0.8)	0.6		0.9 (0.7)	0.20		534.3 (41.5)	< 2.0E−16	
Fiber type		<2.2E-16			7.40E−05			< 2.2E−16			2.80E−07			0.0002			4.10E−14			< 2.0E−16	
Oligosaccharides	Ref.		255.7	Ref.		75.1	Ref.		156.3	Ref.		1.9	Ref.		1.9	Ref.		2.5	Ref.		532
Blank	−7.3 (0.4)		63.3	−0.4 (0.1)		39.3	−7.9 (0.5)		33.7	0.4 (0.1)		3.3	0.4 (0.1)		3.0	0.8 (0.1)	< 0.0001	6.4	−360.0 (17.1)		164
RS	−3.7 (0.4)		126.6	−0.3 (0.1)		51.4	−4.3 (0.5)		84.9	0.1 (0.1)		2.4	0.6 (0.1)		3.1	0.6 (0.1)	< 0.0001	4.6	−223.8 (17.1)		296
Inulins	−3.3 (0.4)		151.3	0.1 (0.1)		68.5	−0.5 (0.5)		160.3	0.2 (0.1)		2.3	0,2 (0.1)		2.2	0.1 (0.1)	0.35	2.9	−106.4 (17.1)		418
PD diagnosis		0.93			0.09			0.05			0.88			0.790			0.77			0.81	
Healthy volunteers	Ref.		152.2	Ref.		47.8	Ref.		122.4	Ref.		2.3	Ref.		2.3	Ref.		4.0	Ref.		364
Parkinson’s patients	0.1 (1.0)		130.2	0.6 (0.4)		67.5	−3.5 (1.8)		81.5	0.1 (0.6)		2.6	0.2 (0.8)		2.7	-0.2 (0.7)		3.6	−10.0 (42.8)		341.0
Sex		0.01			0.03			0.81			0.58			0.80			0.84			0.91	
Female	Ref.		111.1	Ref.		85.0	Ref.		96.6	Ref.		1.9	Ref.		2.2	Ref.		3.5	Ref.		350
Male	2.7 (1.0)		174.5	−0.8 (0.4)		37.9	0.4 (1.8)		105.3	0.4 (0.6)		3.0	0.2 (0.8)		2.8	0.2 (0.8)		4.1	4.8 (42.6)		355.0
Fiber type * PD diagnosis		0.01			0.003			0.003			0.02			0.23			0.36			0.82	
Blank * PD diagnosis	−1.4 (0.6)			−0.6 (0.2)			2.4 (0.7)			−0.01 (0.1)			0.1 (0.2)			0.3 (0.2)			−15.0 (42.2)		
RS * PD diagnosis	−2.0 (0.6)			−0.1 (0.2)			2 (0.7)			0.2 (0.1)			−0.2 (0.2)			0.1 (0.2)			−23.0 (42.2)		
Inulins * PD diagnosis	−0.7 (0.6)			−0.4 (0.2)			1.3 (0.7)			−0.2 (0.1)			−0.2 (0.2)			0.1 (0.2)			−14.4 (42.2)		

C4. SCFA~ Fiber type * PD diagnosis + Sex + (1|ID)																					
Intercept	13.8 (1.3)	< 0.0001		4.3 (0.3)	< 2.2E−16		13.1 (1.7)	< 0.0001		1 (0.6)	0.09		0.2 (0.8)	0.8		0.5 (0.7)	0.45		479.5 (51.9)	< 2.0E−16	
Fiber type		< 2.2E−16			0.0002			< 2.2E−16			1.10E−09			3.2E−15			< 2.0E−16			< 2.0E−16	
Oligosaccharides	Ref.		238.2	Ref.		65.1	Ref.		148.5	Ref.		1.6	Ref.		1.7	Ref.		2.0	Ref.		493
Blank	−6.4 (0.4)		68.0	−0.1 (0.1)		41.6	−7.2 (0.6)		35.1	0.7 (0.1)		3.6	0.8 (0.1)		3.5	1.4 (0.1)	< 0.0001	7.4	−303.9 (20.1)		174
RS	−2.9 (0.4)		133.9	−0.1 (0.1)		58.0	−3.1 (0.6)		95.4	0.3 (0.1)		2.7	0.9 (0.1)		3.3	1.1 (0.1)	< 0.0001	5.1	−158.3 (20.1)		325
Inulins	−2.3 (0.4)		155.4	0.4 (0.1)		72.0	1.3 (0.6)		181.6	0.4 (0.1)		2.3	0.5 (0.1)		2.3	0.5 (0.1)	< 0.0001	2.9	−12.0 (20.1)		451
PD diagnosis		0.91			0.02			0.10			0.76			0.58			0.86			0.97	
Healthy volunteers	Ref.		155.6	Ref.		47.5	Ref.		128.7	Ref.		2.3	Ref.		2.3	Ref.		3.9	Ref.		373
Parkinson’s patients	0.1 (1.3)		129.6	0.7 (0.3)		70.8	−2.8 (1.7)		87.1	0.2 (0.6)		2.7	0.4 (0.8)		2.8	0.1 (0.7)		3.8	2.1 (53.3)		348
Sex		0.02			0.005			0.58			0.58			0.80			0.81			0.65	
Female	Ref.		107.8	Ref.		90.1	Ref.		97.3	Ref.		2.0	Ref.		2.3	Ref.		3.5	Ref.		349
Male	3.1 (1.3)		181.6	−0.9 (0.3)		37.3	0.9 (1.7)		116.9	0.3 (0.6)		3.1	0.2 (0.8)		2.8	0.2 (0.7)		4.2	24.3 (53.5)		373
Fiber type * PD diagnosis		0.01			0.0001			0.05			0.29			0.03			0.38			0.21	
Blank * PD diagnosis	−1.7 (0.6)			−0.6 (0.2)			1.9 (0.9)			−0.1 (0.1)			−0.1 (0.2)			−0.1 (0.2)			−30.0 (28.4)		
RS * PD diagnosis	−2.0 (0.6)			−0.1 (0.2)			1.4 (0.9)			0.1 (0.1)			−0.4 (0.2)			−0.2 (0.2)			−18.2 (28.4)		
Inulins * PD diagnosis	−1.3 (0.6)			−0.5 (0.2)			−0.04 (0.9)			−0.1 (0.1)			−0.4 (0.2)			−0.2 (0.1)			−59.2 (28.4)		

C5. SCFA~ Fiber type * PD diagnosis + Sex + (1|ID)																					
Intercept	13.9 (1.2)	< 0.0001		4.3 (0.3)	< 2.2E−16		13.3 (1.6)	< 0.0001		1.1 (0.6)	0.09		0.2 (0.8)	0.8		0.8 (0.7)	0.28		486.8 (45.7)	< 2.0E−16	
Fiber type		< 2.2E−16			1.03E−05			< 2.0E−16			3.50E−08			1.3E−15			< 2.0E−16			< 2.0E−16	
Oligosaccharides	Ref.		254.5	Ref.		70.4	Ref.		157.3	Ref.		1.7	Ref.		1.7	Ref.		2.4	Ref.		526
Blank	−5.2 (0.5)		83.1	−0.2 (0.2)		46.7	−7.4 (0.7)		41.6	0.7 (0.1)		4.4	0.8 (0.1)		4.2	1.3 (0.1)		9.3	−287.2 (24.2)		205
RS	−2.7 (0.5)		138.1	−0.1 (0.2)		58.5	-2.6 (0.7)		111.8	0.4 (0.1)		3.1	1 (0.1)		3.8	1 (0.1)		6.1	−137.2 (23.5)		348
Inulins	−1.6 (0.5)		174.2	0.5 (0.2)		85.7	0.9 (0.7)		207.0	0.4 (0.1)		2.7	0.6 (0.1)		2.5	0.5 (0.1)		3.4	16.3 (23.5)		517
PD diagnosis		0.240			0.03			0.090			0.87			0.61	3.2		0.92			0.26	
Healthy volunteers	Ref.		166.0	Ref.		52.1	Ref.		135.3	Ref.		2.7	Ref.		2.6	Ref.		4.9	Ref.		397
Parkinson’s patients	1.4 (1.2)		147.0	0.7 (0.3)		78.0	−2.7 (1.6)		107.0	0.1 (0.6)		3.2	0.4 (0.8)			−0.1 (0.8)		4.4	54.2 (47.9)		402
Sex		0.02			0.005			0.42			0.55			0.73			0.83			0.61	
Female	Ref.		123.8	Ref.		99.2	Ref.		107.2	Ref.		2.3	Ref.		2.5	Ref.		4.2	Ref.		387
Male	2.8 (1.2)		192.7	−0.9 (0.3)		40.9	1.3 (1.6)		135.1	0.4 (0.6)		3.6	0.3 (0.8)		3,3	0.2 (0.8)		5.1	23.4 (46.1)		411
Fiber type * PD diagnosis		1.50E−05			0.009			0.06			0.05			0.001			0.32			0.08	
Blank * PD diagnosis	−3.2 (0.7)			−0.5 (0.2)			2.7 (1.0)			0.2 (0.2)			0.1 (0.2)			0.1 (0.2)			−66.7 (34.3)		
RS * PD diagnosis	−3.1 (0.7)			−0.1 (0.2)			1.2 (1.0)			0.2 (0.2)			−0.4 (0.2)			−0.01 (0.2)			−80.3 (33.2)		
Inulins * PD diagnosis	−2.3 (0.7)			−0.7 (0.2)			1.9 (1.0)			−0.2 (0.2)			−0.5 (0.2)			0.2 (0.2)			−48.9 (33.2)		

C6. SCFA~ Fiber type * PD diagnosis + sex + (1|ID)																					
Intercept	13.7 (1.3)	< 0.0001		4.3 (0.3)	< 2.2E−16		13.3 (1.6)	< 0.0001		1.0	0.11		0.2 (0.7)	0.73		0.8 (0.7)	0.22		486.5 (51.8)	< 2.2E−16	
Fiber type		< 2.2E−16			0.002			< 2.2E−16			1.00E−11			< 2.2E−16			< 2.0E−16			<2.2E−16	
Oligosaccharides	Ref.		241.6	Ref.		70.6	Ref.		158.2	Ref.		1.8	Ref.		1.8	Ref.		2.4	Ref.		519
Blank	−4.3 (0.6)		89.5	0.004 (0.1)		49.4	−7.0 (0.9)		43.2	1 (0.1)		5.0	1 (0.1)		4.9	1.5 (0.1)		9.7	−246.6 (23.6)		220
RS	−2.0 (0.6)		154.3	−0.03 (0.1)		63.1	−1.8 (0.8)		126.2	0.5 (0.1)		3.5	1.1 (0.1)		4.2	1.1 (0.1)		7.0	−97.7 (22.8)		387
Inulins	−1.2 (0.6)		193.2	0.5 (0.1)		85.6	1.6 (0.9)		236.2	0.5 (0.1)		2.7	0.4 (0.1)		2.3	0.2 (0.1)		3.0	40.2 (23.6)		555
PD diagnosis		0.500			0.04			0.080			0.68			1.00E−04			92			0.44	
Healthy volunteers	Ref.		174.6	Ref.		54.6	Ref.		150.2	Ref.		2.8	Ref.		2.7	Ref.		5.0	Ref.		422.4
Parkinson’s patients	0.9 (1.3)		155.0	0.7 (0.3)		79.5	−3.0 (1.7)		112.8	0.3 (0.6)		3.4	0.5 (0.7)		3.4	−0.1 (0.7)		4.4	41.7 (54)		418
Sex		0.03			0.008			0.36			0.59			0.58			0.81			0.65	
Female	Ref.		131.0	Ref.		103.7	Ref.		114.3	Ref.		2.5	Ref.		2.7	Ref.		4.3	Ref.		409
Male	2.8 (1.3)		202.1	−0.9 (0.3)		41.9	1.5 (1.6)		148.5	0.4 (0.7)		3.8	0.2 (0.8)		3.4	0.2 (0.7)		5.1	23.8 (53)		432
Fiber type * PD diagnosis		7.00E−04			0.002			0.20			0.32			1.00E−03			0.81			0.004	
Blank * PD diagnosis	−3.5 (0.9)			−0.7 (0.2)			2 (1.2)			−0.1 (0.2)			−0.04 (0.1)			−0,2 (0.2)			−105.1 (33.3)		
RS * PD diagnosis	−2.2 (0.9)			−0.2 (0.2)			1 (1.2)			0.02 (0.2)			−0.5 (0.1)			−0.1 (0.2)			−68.2 (33.3)		
Inulins * PD diagnosis	−1.0 (0.9)			−0.5 (0.2)			2.4 (1.2)			−0.3 (0.2)			−0.4 (0.2)			−0.02 (0.2)			−9.3 (34.4)		

C7. SCFA~ Fiber type * PD diagnosis + Sex + (1|ID)																					
Intercept	14.2 (1.4)	< 0.0001		4.4 (0.4)	< 2.2E−16		13.5 (1.5)	< 0.0001		1.1 (0.6)	0.09		0.4 (0.7)	0.58		0.8 (0.7)	0.23		505.5 (51.2)	< 2.0E−16	
Fiber type		< 2.2E−16			0.11			< 2.2E−16			7.20E−11			8.4E−10			< 2.0E−16			< 2.0E−16	
Oligosaccharides	Ref.		249.4	Ref.		72.8	Ref.		165.0	Ref.		1.9	Ref.		1.9	Ref.		2.4	Ref.		529
Blank	−4.6 (0.6)		91.9	0.1 (0.2)		53,7	−7.0 (0.9)		45.3	1.2 (0.2)		6.2	0.9 (0.2)		5.3	1.7 (0.2)		11.6	−247.4 (25.6)		238
RS	−1.9 (0.6)		161.2	−0.01 (0.2)		68.2	−1.1 (0.9)		137.3	0.5 (0.2)		3.6	0.9 (0.2)		4.3	1.1 (0.2)		7.6	−77.9 (25.6)		412
Inulins	−1.4 (0.6)		179.0	0.4 (0.2)		81.5	1.4 (0.9)		227.9	0.3 (0.2)		2.5	0.3 (0.2)		2,4	0.3 (0.2)		3.1	23.6 (25.6)		547
PD diagnosis		0.740			0.07			0.08			0.77			0.80			0.86			0.82	
Healthy volunteers	Ref.		184.0	Ref.		58.5	Ref.		158.3	Ref.		3.5	Ref.		3.0	Ref.		5.6	Ref.		447
Parkinson’s patients	0.5 (1.4)		147.8	0,7 (0.4)		79.8	-2.8 (1.6)		112.6	0.2 (0.6)		3.6	0.2 (0.7)		3.4	−0.1 (0.7)		4.6	12.2 (53.4)		415.0
Sex		0.05			0.02			0.29			0.56			0.72			0.69			0.51	
Female	Ref.		131.5	Ref.		104.5	Ref.		116.4	Ref.		2.7	Ref.		2.8	Ref.		4.4	Ref.		414
Male	2.8 (1.4)		203.2	−0.9 (0.4)		44.6	1.6 (1.5)		154.0	0.4 (0.7)		4.1	0.3 (0.8)		3.6	0.3 (0.7)		5.8	34.5 (51.9)		448
Fiber type * PD diagnosis		5.00E−04			0.003			0.33			0.59			0.26			0.38			0.02	
Blank * PD diagnosis	−3.1 (0.8)			−0.8 (0.2)			1.7 (1.3)			−0.2 (0.2)			0.2 (0.2)			−0.4 (0.2)			−88.0 (35.7)		
RS * PD diagnosis	−2.4 (0.8)			−0.1 (0.2)			−0.007 (1.3)			0.1 (0.2)			−0.3 (0.2)			0.04 (0.2)			−78.6 (35.7)		
Inulins * PD diagnosis	−2.1 (0.8)			−0.5 (0.2)			1.7 (1.3)			−0.1 (0.2)			−0.2 (0.2)			−0.06 (0.2)			−11.6 (35.7)		

	*Clostridium Leptum*			*Clostridium Coccoides*		
	Estimate (SD)	*P*-value	LSM	Estimate (SD)	*P*-value	LSM
**D. Clostridium abundance~ Fiber type + PD diagnosis + Sex + (1|ID)**						
Intercept	−1.3 (0.07)	< 2.0E−16		−1.52 (0.1)	< 2.0E−16	
Fiber type		5.00E−04			3.20E−16	
Blank	Ref.		−1.4	Ref.		−1.6
Inulins	0.06 (0.03)		−1.3	0.003 (0.03)		−1.6
Gums	0.08 (0.03)		−1.3	0.21 (0.03)		−1.4
Oligosaccharides	0.04 (0,02)		−1.3	0.12 (0.02)		−1.5
Pectins	0.1 (0.03)		−1.3	0.15 (0.03)		−1.5
RS	−0.01 (0.03)		−1.4	0.14 (0.02)		−1.5
Rest group	−0.06 (0.04)		−1.4	0.19 (0.03)		−1.4
PD diagnosis		0.02			0.042	
Healthy volunteers	Ref.		−1.2	Ref.		−1.3
Parkinson’s patients	−0.30 (0.12)		−1.5	−0.33 (0.16)		−1.7
Sex		0.44			0.43	
Female	Ref.		−1,4	Ref.		−1.6
Male	0.09 (0.12)		−1.3	0.12 (0.15)		−1.4

*LSM* per factor averaged out over other factors’ levels. *IDF* insoluble dietary fiber, *LSM* Least square means, norm. gene abund., normalized gene abundance, *PD* Parkinson’s disease, *RD* resistant dextrin, *Ref*. reference, *RS* resistant starch, *SCFA* short chain fatty acids, *SD* standard deviation, *SDF* soluble dietary fiber. The rest group (A) includes cellulose, hemicellulose and lignin. Results are based on fermentation experiments in samples from 19 Parkinson’s disease patients and 35 healthy controls (A), 12 Parkinson’s disease patients and 10 healthy controls (B), 3 Parkinson’s disease patients and 3 healthy controls (C1-7), and from 16 Parkinson’s disease patients and 25 healthy controls (D). Estimates of acetic acid, butyric acid and isobutyric acid are shown as √(μmol/g), estimates of propionic acid, valeric acid and isovaleric acid are shown as log(μmol/g). Estimates of Total SCFA and LSM are shown as μmol/g. Estimates and LSM of both *Clostridium* groups are shown as log (gene abundance).

Visual inspection of Fig. 1 suggested PD patients had a higher mean valerate production after fermentation with fiber supplements than HC. In 21% of PD patients, valerate increased between 1.3 and 27 times the valerate concentration in blanks after fermentation with the majority of fiber supplements tested in those PD patients’ samples. However, multivariate analysis showed no effect of PD diagnosis on valerate production.

**Fig. 3:**
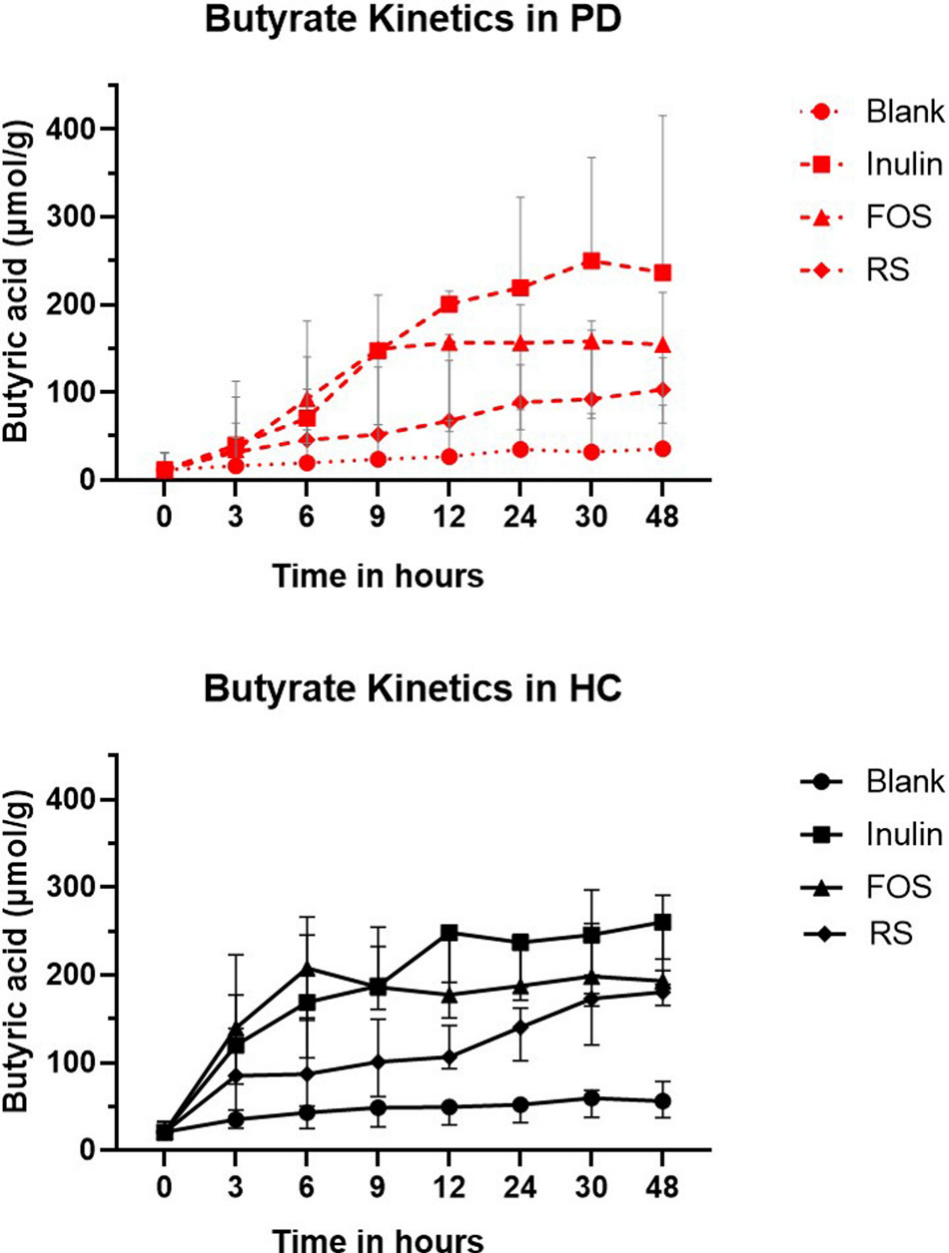
Kinetics of butyrate production during 48h fermentation of Orafti HP, Actilight P950 and Novelose 330 in Parkinson’s disease patients and healthy controls. Results are shown as median ± range, PD Parkinson’s disease patients, HC healthy controls, Orafti HP is inulin; Actilight P950 is FOS; Novelose 330 is resistant starch. Butyrate kinetics were analyzed using linear mixed models per time point, sex, fiber type, PD diagnosis and interaction effect of fiber type, and PD diagnosis were added as fixed factors, the participant was added as a random factor. The kinetic profile indicated that 3–12 h after inulin, RS, and FOS fermentation, PD had lower butyrate production compared to HC.

The kinetic profile indicated that 3–12 h after inulin, RS, and FOS fermentation, PD had lower butyrate production compared to HC, see Fig. 3 and Table 2C^1–7^. The post-hoc analysis demonstrated only a trend towards a difference between PD and HC in butyrate production after 3 h FOS fermentation (*p* = 0.06). The area under the curve (AUC) after fermentation with all 3 fibers was higher in HC compared to PD, although the difference was not statistically significant (Supplementary Table 4). No significant differences in C_max_ or t_max_ were observed between PD patients and HC, for inulin C_max_ was almost the same between PD patients and HC (236.1 ± 95.4 µmol/g vs. 236.0 ± 19.9 µmol/g) (Supplementary Table 4). No differences in pH were observed between PD patients and HC (Supplementary Table 5).

The next step was to investigate the effect of fiber fermentations on butyrate-producers. Univariate analysis showed a negative effect and a negative trend of PD on *C. coccoides* (*p* = 0.03) and *C. leptum* (*p* = 0.09) abundances respectively, when only taking post-incubation blanks into account. This may explain the lower AUC of SCFA in PD (see Fig. 3). The potential effect of PD medication, disease duration, and antidepressant intake on the abundance of butyrate-producers was analyzed within PD patients. No associations were found. Fiber type and PD diagnosis significantly influenced *C. coccoides* and *leptum* abundances, see Table 2D. Only pectin (*p* = 0.02) increased *C. leptum* abundance differently from blanks. Pectin resulted in higher *C. leptum* abundance compared to RS (*p* = 0.008) and fibers consisting of cellulose, hemicelluloses, and lignin (*p* = 0.006). No significant difference was found between other fiber types. All fiber types (*p* < 0.0001), except inulins, increased *C. coccoides* abundance. Results of *Clostridium*-group abundances in PD and HC are shown in Supplementary Fig. 9.

### Vegetable and quinoa fibers

Univariate analyses of baseline SCFA concentrations prior to fermentation (Blank T0) demonstrated a trend (*p* = 0.07) towards lower acetate concentration in PD patients compared to HC. Univariate analyses of vegetable and quinoa fibers fermentation experiments demonstrated overall reduced production of acetate (*p* = 0.01) and total SCFA (*p* = 0.03) in PD compared to HC, whereas a trend (*p* = 0.08) of lower butyrate production was found. Further univariate analyses are shown in Supplementary Table 2. These experiments demonstrated no associations between PD medication or antidepressant intake and SCFA production within PD patients. A trend (*p* = 0.06) towards a positive association between disease duration and isovalerate was found.

Multivariate models of vegetable and quinoa fermentation experiments also demonstrated PD diagnosis and fiber type to be the strongest predictors of SCFA production, see Table 2B. Soluble dietary fiber (SDF) stimulated butyrate production more (*p* < 0.0001) compared to insoluble dietary fiber (IDF) (see Fig. 1). Not only inulin-rich SDF substrates increased butyrate production greatly, but also oyster mushroom stem SDF, rich in β-glucans, stimulated butyrate production considerably in PD and HC, see Supplementary Table 6 and Supplementary Fig. 10. Overall acetate production was significantly reduced in PD, while fiber stimulated acetate production. Fiber stimulated total SCFA production, whereas a trend of overall reduced production in PD was found. Multivariate analysis demonstrated no significant effect of PD on butyrate production in these experiments.

## Discussion

According to the authors’ knowledge, this is the first study to assess dietary fiber’s effect on fecal SCFA production of PD patients. Our results demonstrated that all fiber types stimulated SCFA production, in both PD and HC. However, PD diagnosis limited SCFA production and negatively influenced both *Clostridium*-group abundances. We only report an overall significant difference in SCFA production between PD patients and HC, and not a significant difference in SCFA production between PD patients and HC per fiber type. Interaction effects between PD and fiber type were not considered because no interaction was observed after visualization, furthermore, the consequent multiple post-hoc analyses would increase bias because of multiple testing.

Large inter-individual variability in SCFA production was observed in both groups. High inter-individual variability in gut microbiota responses to dietary interventions has been reported^31–33^. Studies suggest that dietary habits and baseline gut microbiome compositions influence inter-individual variability^32^. Participants’ dietary habits were not evaluated in our study, only dietary fiber intake of the day prior to sampling. Therefore, we cannot conclude if diet or other factors are the main cause of the inter-individual variation.

Still, both fermentation experiments found similar results regarding the limited SCFA production capacity of PD patients compared to HC. Unger et al. (2016) found reduced fecal acetate, propionate, and butyrate concentrations in PD patients, compared to age-matched controls^5^. In contrast, our results demonstrated no influence of PD on propionate production. Our results however include the influence of fiber fermentation, whereas Unger et al. (2016) only investigated basal SCFA concentrations^5^. Our results demonstrated a significant lower basal acetate concentration and a trend towards lower butyrate concentration in the PD patients compared to HC included in the fiber supplement experiments. In the vegetable and quinoa fibers experiments, a trend towards lower basal acetate concentration in PD patients compared to HC was found. These discrepancies in our results and those of Unger et al. (2016) might be explained by the lower number of participants in our study or potential differences in dietary intake.

A positive association between COMT inhibitors and total SCFA was found in the fiber supplement, but not in vegetable and quinoa fiber experiments. In the latter experiments however, a trend (*p* = 0.05) towards a positive association between COMT inhibitors and butyrate production was found. This is in contrast with findings of Unger et al. (2016)^5^ and a recent pilot study by Grün et al. (2020), that found a negative association between entacapone, but not other COMT inhibitors, and *Faecalibacterium prausnitzii* (a butyrate-producer) and a trend towards reduced fecal butyrate^34^. It is not clear how COMT inhibitors are associated with SCFA production. Studies by Unger et al. (2016) and Grün et al. (2020) are based on relatively small sample size and also in our study only 7 PD patients reported COMT inhibitor intake. Another discrepancy between our study and the studies by Unger et al. (2016) and Grün et al. (2020) is that in our study SCFA production was stimulated in vitro, whereas both Unger et al. (2016) and Grün et al. (2020) investigated the basal butyrate concentration in fecal samples. The latter refers to butyrate that is not absorbed by the colonocytes in vivo and is consequently excreted. In vivo, COMT inhibitors may be associated with higher SCFA production, which in result may also lead to increased absorption. Further studies are needed to elucidate the potential association between COMT inhibitors and SCFA production.

A negative association and negative trend of levodopa between isobutyrate and isovalerate production were observed in our study. Isobutyrate and isovalerate are protein fermentation end products^35^. Due to food-drug interaction between dietary amino acids and levodopa, PD patients are advised not to combine levodopa and protein intake^36^. This may explain the observed association.

Inulins had the largest effect on SCFA production in both PD and HC, which is consistent with literature^37,38^. Koecher et al. (2014) reported similar mean total SCFA production after 24 h inulin fermentation in HC aged 24–32 years to our results (643 ± 59 mmol/l versus our 560.3 ± 17.7 µmol/g in PD and 592.5 ± 21.9 µmol/g in age-matched HC, reported as mean ± SEM). Although RS is currently considered the most butyrogenic substrate^25,39^, our results indicated a lesser effect of RS compared to inulins, confirming previous studies^38,40^. This may be due to RS’ lower fermentation rate^25^, as also indicated by our kinetics experiment.

PD diagnosis negatively influenced relative *C. coccoides* and C. *leptum* abundances, consistent with reported lower abundances of butyrate-producers^2–5,7,41^. This is probably part of the mechanism behind PD diagnosis’ limiting effect on SCFA production. In contrast to our results, Qian et al. (2018) found an increase in *C. leptum* abundance in PD patients^42^. No information regarding dietary fiber intake was provided, however differences in fiber intake may explain this inconsistency. Our study clearly shows that fiber supplements significantly influenced *Clostridium*-group abundances in vitro, which is consistent with other studies^43–45^. Though inulins resulted in the largest butyrate increase, inulins were not associated with either *Clostridium*-group abundances. This suggests that increased butyrate production following inulin fermentation may be an effect of cross-feeding^46^.

Kinetics showed fiber fermentation resulted in lower butyrate production in PD compared to HC during the first 12 h, potentially due to reduced butyrate-producers. At 48 h butyrate production of PD was similar to HC, indicating that PD patients’ remaining butyrate-producers still ferment fiber, but with a slower production start. Remarkably, C_max_ of inulin fermentation was similar in PD and HC, whereas the AUC of all fibers was higher in HC. The effect of PD diagnosis on AUC was not statistically significant, probably because of the high variability in SCFA production and the limited sample size of the kinetics experiments. Increased daily fiber intake (depending on fiber type) may activate butyrate-producers in PD patients, thereby increasing the fermentation rate. This could not be confirmed by dietary fiber intake, however, those results are based on one day and may not accurately represent usual fiber intake. Brahma et al. (2017) demonstrated that gut microbiota from people on a high-quality diet (characterized by high fiber intake) were more equipped for butyrate production compared to those of people with a lower quality diet^47^. Longer reporting periods of dietary fiber intake and the association with butyrate production in PD patients would be interesting to investigate further.

Vegetable-derived SDF increased butyrate production more than IDF in both PD and HC, which could be explained by solubility or by fiber composition^48,49^. Belgian endive roots, chicory roots, and black salsify are inulin-rich^50–52^, which also demonstrated butyrogenic effects in PD and HC in fiber supplement experiments. Oyster mushrooms are rich in β-glucans^53^, hemicellulose that has bifidogenic properties and influences SCFA production^54^. Consistent with our results, fungal β-glucans are promising prebiotic candidates, which were shown to increase *Faecalibacterium prausnitzii* and butyrate in fecal samples of HC older than 65 years^55^. Vegetable and quinoa IDF fractions are rich in hemicelluloses, cellulose, and lignin^56–61^. Cellulose is generally not completely fermented in the gut, because of its structure^49^, potentially explaining the lower butyrate production of IDF.

The current study has some limitations. A static fermentation model was used, which is effective for the fermentation extent/rate assessment of dietary fibers and their SCFA production, however it is limited since it does not consider SCFA absorption. Recruitment of PD patients was difficult, participants often did not meet the in- or exclusion criteria or were unwilling to participate, and the amount of fecal sample collected by participants was often (too) small, thereby limiting the number of fibers to be tested and the number of analyses that could be carried out. The ratio of men to women were significantly different between PD patients and HC. Although sex was included as a potential confounding variable in the linear mixed models, this still may have impacted our results. The use of steroids was not used as an exclusion criterion, although steroid use has been reported to impact the gut microbiome. However, only 1 HC reported the use of steroids, therefore the impact on our results should be limited. Though the kinetics results provide an interesting first look into PD patients’ butyrate production, they are based upon limited sample size, thereby limiting external validity. Nonetheless, this study provides a useful first insight into dietary fiber’s effect on SCFA production in PD patients. Current findings, however, do not allow to provide any dose-response relationship beneficial for PD patients. In future studies, it would be interesting to validate these findings in colonic mucosal samples and to use a more comprehensive approach to gut microbiota characterization through the use of next-generation sequencing, proteomics, and pathway analysis. Furthermore, reporting results by specific PD phenotypes may have added value, as it has been reported that the gut microbiome differs between different clinical PD phenotypes^7^.

To conclude, this study demonstrates that dietary fiber stimulates butyrate production in PD patients despite decreased butyrate-producing bacteria. However, the butyrate production remains reduced compared to HC. Inulins in contrast to RS increase butyrate production most in both PD and HC, however, the SCFA production start is indicated to be slower in PD compared to HC. Of the vegetables, both the inulin-rich vegetables as the β-glucan-rich oyster mushrooms demonstrated butyrogenic effects. Dietary fiber intake may be a promising approach in PD, but further in vivo research is needed to investigate increased fiber intake’s effect on plasma SCFA levels and motor symptoms.

## Methods

### Study design

An in vitro study was conducted between November 2018 and November 2019, in fecal samples of PD patients and HC of similar age. The study consisted of fecal sample collection for fermentation experiments, a questionnaire about general health (including weight loss evaluation, based on NRS 2002 methodology^62^) and medication intake and a fiber intake screening questionnaire based on the day before sample collection^63^. In accordance with the advice of the Ethics Committee of the University of Leuven, participant data was anonymized, therefore no written informed consent was obtained. However, all participants provided oral informed consent prior to participating in the study. The study protocol complied with the Helsinki declaration and was approved by the Ethics Committee of the University of Leuven (11^th^ of October 2018–Reference B322201837674 – S61782). To evaluate SCFA production in PD, a stepwise approach was used. First fermentation experiments were performed using fiber supplements, second SCFA production kinetics during fermentation with three fiber types was examined and third fibers’ effects on two butyrate-producing bacterial groups were assessed. Finally, to evaluate vegetables’ potential, fermentation experiments were performed with vegetable-derived fibers.

### Study population

PD patients and HC (men and postmenopausal women) were recruited in collaboration with a regional hospital (AZ Sint-Jan - Bruges) and regional departments of the patient’s organization ‘Flemish Parkinson Association’. Inclusion criteria were age between 55 and 70 and BMI between 18.5–25 kg/m², for PD patients idiopathic Parkinson’s disease diagnosis was added. Exclusion criteria were antibiotics use or use of pre- and probiotics for 3 months prior to the study, prior gastrointestinal surgery, diagnosis of atypical or secondary parkinsonism or gastrointestinal diseases, including Crohn’s disease, colorectal cancer, and colitis ulcerosa.

### Pretreatment

All fiber supplements, selected vegetables, quinoa varieties, and their sources used in the fermentation experiments are listed in Supplementary Table 7. Vegetables were acquired fresh, except kale (freshly frozen) and black salsify (blanched and cooled). They were comminuted before air-drying at 60 °C. Quinoa and dried vegetables were ground using an Ultracentrifugal Mill (Retsch, Germany) with a 750 µm sieve before purification. All fiber substrates underwent purification prior to fermentation, to remove most proteins and mono- and disaccharides present, according to Dalgetty and Baik (2003), with some modifications^64,65^. Purification resulted in a SDF and/or IDF substrate. Substrates underwent a water-based fractionation into a soluble and insoluble fraction, when applicable. In the soluble fraction, proteins were precipitated by pH adjustment from pH 3 to pH 9, and mono- and disaccharides were removed through nanofiltration using the Alfa Laval LabStak™ M20 module (Alfa Laval, Sweden)^64–67^. Membranes (Alfa Laval, Sweden) had a 300 Dalton molecular weight cut-off. Purified soluble fractions were stored at −20 °C before freeze-drying. The insoluble fraction obtained after water-based fractionation was, when present, wet-screened through sieves ranging from 56–710 µm^64^. The collected sediment was treated with alpha-amylase (MATS L Classic, > 7400 Thermostable α-amylase units per gram, IMCD, The Netherlands) at 70 °C during 30 min^65^. After centrifugation, the residue was collected and stored at −20 °C before freeze-drying. Freeze-dried powders were sterilized using gamma sterilization (dose of 15kGy) carried out by Synergy Health, the Netherlands.

To control purification, soluble carbohydrates were analyzed in fiber supplements, vegetable, and quinoa fiber substrates^68,69^. Only in Belgian endive roots, black salsify, chicory roots, and oyster mushroom stems, SDF was purified since most vegetables and all quinoa samples had an SDF concentration equal to or below mono- and disaccharides concentration, which complicated purification. Vegetable and quinoa substrates were analyzed regarding polyphenol^70,71^, protein^72^, and starch content (Megazyme Digestible and Resistant Starch Assay Kit, Megazyme, Ireland) and anti-oxidative capacities^73–75^. Results are shown in Supplementary Tables 8–10.

### Fermentation

All fiber substrates used in fermentation experiments are shown in Supplementary Table 7. Each substrate was evaluated in a minimum of 8 fermentation experiments (fecal samples of min. 4 PD patients and 4 HC), see Supplementary Table 1. Fiber supplement fermentations were carried out in samples of 19 PD patients and 35 HC. For vegetable and quinoa fiber fermentation, samples of 12 PD patients and 10 HC were used, of which respectively 7 and 6 were also included in the fiber supplements experiment. Fecal samples were collected in recipients containing Oxoid AnaeroGen 3.5 L Sachet (Thermo Fisher Scientific, USA) and stored at 4 °C. Participants were requested to report the date and time of sampling, time between sampling and last defecation and Bristol Stool Form Scale (BSFS)^76^. Sample collection was based on previously published studies^77–79^. The use of Oxoid Anaerogen during sample collection induces an anaerobic environment (oxygen level below 1%) in the sample recipient as soon as it is closed, which has been demonstrated to maintain the viability of > 90% of the extremely oxygen-sensitive gut microbiota^80^

Fecal samples were transported to the lab on ice within 24 h after collection. In the lab, the sample was introduced in an anaerobic cabinet (Whitley A35 Workstation, Don Whitley Scientific, UK) and homogenized with anaerobic phosphate-buffered saline (Thermo Fisher Scientific, USA) (1 in 10 dilution) into a fecal slurry. Aliquots of 5 ml slurry were made, dietary fibers (1% w/v) were added and anaerobically incubated during 24 h at 37 °C. Slurry without fiber was incubated as a negative control.

Each incubation was done in triplicate. After incubation, samples were placed on ice to cease fermentation and stored at −80 °C until further analysis. In a subset of participants, slurry samples without fiber were collected before incubation and stored at −80 °C until analysis. These samples were used to assess baseline SCFA concentration.

Fermentation kinetics were studied using inulin, FOS, and RS in anaerobic fermentation during 48 h, with 8 sampling points: baseline, 3, 6, 9, 12, 24, 30, and 48 h. Inulin, FOS, and RS were chosen because these fibers have already been extensively studied and are known for their butyrogenic effects^81^. Kinetics of these 3 fibers were investigated using fecal samples of 3 PD patients and 3 HC (for both PD patients and HC, samples were collected from 2 men and 1 woman), following the above-described method. These experiments were carried out to investigate if SCFA of PD patients are produced in the same velocity and quantities as in HC.

### SCFA analysis

Standards for SCFA analysis (acetic acid, acetic acid D4, butyric acid, isobutyric acid, isovaleric acid, propionic acid, valeric acid, and valeric acid D9) were purchased from Sigma Aldrich, USA. Formic acid was purchased from Biosolve, the Netherlands.

For analysis of acetate, propionate, butyrate, valerate, isobutyrate, and isovalerate in fecal slurries, samples were prepared as follows. Of the slurry, 200 µl was added to a 20 mL headspace vial (Research Institute for Chromatography (RIC), Belgium). Also, 2 g of NaCl, 100 µl of internal standards (IS) acetic acid-D4 (0.1 mmol/ml), and valeric acid-D9 (0.1 mmol/ml) were added. Deionized water containing 0.1% formic acid was added until a total volume of 10 ml was obtained, afterwards the vial was capped. Quantification was done based on relative areas (using IS) and using external standard curves of reference analytical standards. Total SCFA was determined as the sum of all SCFA.

SCFA were extracted and analyzed using automated headspace solid-phase microextraction – gas chromatography-mass spectrometry with a Gertsel MPS sampler coupled to an Agilent 8890GC and 5977B GCMSD (Agilent, USA). After sample preparation, the vial was incubated for 10 min at 45 °C and agitated at 250 rpm, followed by 40 min extraction at the same temperature with a Supelco 50/30 µm Divinylbenzene/Carboxen/Polydimethylsiloxane fiber (Supelco, USA). The SPME fiber was desorbed in splitless mode at 250 °C and analytes were separated on an HP-FFAP column (25 m × 0.2 mm × 0.33 µm) (Agilent, USA) using a helium flow rate of 1.6 ml/min. Oven temperature program was set as follows: start at 60 °C, hold for 1 min, then raised to 230 °C at a rate of 10 °C/min and hold for 2 min. Compounds were ionised through electron impact ionization and the mass spectrometer was operated in selected ion monitoring (SIM)/SCAN mode. Data were processed using the acquired SIM data.

### Quantitative real-time PCR

DNA was extracted from 2 ml slurry using QIAamp Fast DNA Stool mini kit (Qiagen, Germany) according to Knudsen et al. (2016)^82^. *Clostridium leptum* and *Clostridium coccoides* groups, to which many butyrate-producing bacteria belong^83^, were quantified using qPCR in slurries after 24 h fermentation with and without a selection of fiber supplements, see Supplementary Table 7. For this selection, one representative per fiber type was chosen. QPCR was carried out on a Lightcycler 480 Real-time PCR system (Roche, Germany) using SYBR Green. Per fiber, slurries from 5 PD patients and 5 HC were analyzed for the quantification of *Clostridium coccoides* and *Clostridium leptum* groups using qPCR. DNA extracts were eluated using 100 µl elution buffer and DNA concentration was checked using a Quantus fluorometer (Promega Corporation, USA). Per slurry, DNA extracts of two biological replicates were analyzed. Each DNA extract was 100-fold diluted to eliminate PCR-inhibition and analyzed in triplicate. Total 16 S rRNA gene was quantified as a proxy for bacterial load. Previously published primers for *Clostridium* group-specific and eubacterial 16 S rRNA genes were BLASTed and aligned with GenBank sequences to ascertain their location^84–87^. Subsequently, a plasmid (containing eubacterial 16 S rRNA gene target) and a gBlock containing 2 consecutive sequences of interest (a 246 bp sequence of 16 S rRNA of *Clostridium leptum* (consisting of region 914–1159 bp of Genbank accession NR_114789.1) and a 440 bp sequence of 16 S rRNA of *Dorea formicigenerans* (consisting of region 466–905 of Genbank NR_044645.2)) separated by the nucleotides AT, were designed and both obtained from Integrated DNA Technologies, USA. Ten-fold serial dilutions of the plasmid and gblock were used as standards for quantification. SsoAdvanced SYBR green supermix was purchased from Bio-Rad Laboratories, USA. The reaction mixture consisted of 5 µl template DNA, 12.5 µl SsoAdvanced SYBR green supermix, the correct amount of each primer and sterile water to obtain a total volume of 25 µl. For the amplification of *Clostridium* group-specific 16 S rRNA gene-targets, an activation step of 5 min at 94 °C was followed by 30 or 32 cycli (for *Clostridium coccoides* and *leptum*, respectively) of 20 s at 94 °C, 20 s at 50 °C and 15 s at 72 °C and one cycle of 15 s at 94 °C. For the amplification of the eubacterial 16 S rRNA gene target, an activation step of 10 min at 95 °C was followed by 30 cycles of 15 s at 95 °C and 1 min at 60 °C. The specificity of the reaction products was assessed by melting curve analysis. This was performed by gradually increasing the temperature from 60 to 95 °C at a rate of 0.2 °C/s, with continuous fluorescence collection. Details of primer sequences and primer concentration are shown in Supplementary Table 11.

### Data analysis

SCFA data was analyzed using Agilent MassHunter Quantitative Data Analysis (Agilent, USA). QPCR results were analyzed using Lightcycler 480 Software (Roche, Germany). Results of SCFA analysis were corrected for dilution. Relative abundances of *Clostridium* group-specific 16 S rRNA gene targets were calculated by dividing their abundance by total eubacterial 16 S rRNA gene abundance. This normalization was carried out to account for differences in extraction efficiency and total bacterial number.

### Statistical analysis

Possible differences in sex, BSFS and medication intake between PD patients and HC were analyzed using Fisher’s exact test. To evaluate potential differences in BMI, fiber intake and age between PD and HC, Student T-test and Mann–Whitney U-test was used, depending on normality (evaluated by Shapiro Wilk test). Log transformation of both *Clostridium*-group genes, propionate, valerate and isovalerate and square root transformation of acetate, butyrate and isobutyrate was carried out to meet linear regression assumptions (normality, homoscedasticy and independence of residuals, no autocorrelation and little multicollinearity). To investigate factors that may influence *Clostridium*-group abundances or production of the different SCFA, linear mixed models (LMM) were used. Fiber type, PD diagnosis, age, sex, BMI, BSFS, fiber intake, sampling time, time between sampling and last defecation, weight loss, medication intake or *Clostridium*-groups (in SCFA models) were added as fixed factor, participant was used as random factor in univariate analyses. In final multivariate models fiber type, PD diagnosis, sex were added as fixed factors and participant as a random factor. Sex was included as potential confounding variable, due to the imbalance of men/women between PD and HC. No interaction effects were considered in the *Clostridium* or SCFA models. SCFA kinetics were analyzed using LMM per time point, sex, PD diagnosis, fiber type and interaction effect of fiber type and PD diagnosis were added as fixed factors, participant was added as a random factor. Residuals’ normality in all models was assessed using histograms and Q-Q plots, homoscedasticy, and independence of residuals were evaluated by plotting residuals. Post-hoc analysis of blanks (ref) versus fiber types was carried out for LMM of *Clostridium*-groups and SCFA, using Dunnett’s test to correct for multiple testing. Post-hoc pairwise comparisons between beforehand selected fiber types (inulins, oligosaccharides, pectins, RS, and the combination of hemicelluloses, cellulose, and lignin) were carried out, using the Tukey test to correct for multiple testing. The number of post-hoc tests was limited, to reduce the risk of type I errors. Analyses were carried out using RStudio 1.1.456. Statistical significance was determined as *p* < 0.05. Following R packages were used for statistical analyses: emmeans, FSA, GGally, ggplot2, Hmisc, lmtest, lme4, nlme, lmerTest, MuMIn, and psych.

### Reporting summary

Further information on research design is available in the Nature Research Reporting Summary linked to this article.

## Supplementary information

Supplementary Information

Reporting Summary

## Data Availability

The datasets generated and/or analyzed during the current study and the supplementary information are available in the Figshare repository, 10.6084/m9.figshare.13238045.
