# Supplementary material for: Parkinson’s disease patients’ short chain fatty acids production capacity after in vitro fecal fiber fermentation
Source: NPJ Parkinsons Dis. 2021 Aug 13;7:72. doi: 10.1038/s41531-021-00215-5 (PMC8363715; doi:10.1038/s41531-021-00215-5)
Supplement: Supplementary file 1 — Supplementary Information [file 41531_2021_215_MOESM1_ESM.pdf]

Supplementary files

Supplementary Table 1. Number of fecal samples used in fermentation experiments per fiber substrate

Substrates	PD (n)	HC (n)
Oligosaccharides		
Fibrulose F97 - SDF	7	6
Actilight 950P - SDF	9	6
Orafti P95 - SDF	5	5
Human milk oligosaccharide (2'fucosyllactose) - SDF	5	7
GOS - SDF	6	5
Inulins		
Orafti HP - SDF	13	22
Orafti GR - SDF	5	5
Fibruline Instant - SDF	10	14
Fibruline XL - SDF	5	5
Fibruline S30 - SDF	5	5
Frutafit IQ - SDF	5	5
Pectins		
Grindsted Pectin AMD 922 - SDF	7	7
Genu Pectin type B - SDF	5	5
Gums		
Genu gum RL 200 - SDF	5	5
Acacia fiber - SDF	7	6
Xanthan gum - SDF	5	5
Guar gum - IDF	5	5
Guar gum - SDF	5	5
Psyllium - SDF	5	5
Resistent dextrin/starch		
Novelose 330 - IDF	11	10
Promitor - SDF	5	5
Nutriose FM06 - SDF	5	5
Polydextrose		
Litesse Ultra ™ IP Powder - SDF	5	5
Rest group (hemicelluloses, cellulose, lignin)		
Apple fiber - IDF	5	5
Liquid Oat Bran - IDF	5	5
Peafiber I 50M - IDF	5	5
Swelite - Peafiber - IDF	5	5
Sugar beet fiber - IDF	6	5
Sugar beet fiber - SDF	5	5
Vegetable and quinoa substrates		
Belgian endive roots - IDF	5	4
Belgian endive roots - SDF	5	4
Black Salsify - IDF	4	5
Black Salsify - SDF	5	5
Broccoli stems - IDF	4	5
Brussels sprouts - IDF	4	5
Cabbage - IDF	5	4
Chicory roots - IDF	4	4
Chicory roots - SDF	6	5
Kale - IDF	5	5
Oyster mushroom stems - IDF	5	5
Oyster mushroom stems - SDF	5	5
Pointed sweet bell pepper - IDF	5	4
Quinoa Bastille - IDF	5	5
Quinoa Summer Red - IDF	4	5
Quinoa Titicaca - IDF	4	5
White cabbage - IDF	5	4

IDF, insoluble dietary fiber; SDF, soluble dietary fiber; PD, Parkinson's disease patients; HC, healthy controls

Supplementary Figure 1. Boxplot of acetic acid production post fermentation with fiber supplements, vegetable and quinoa fibers

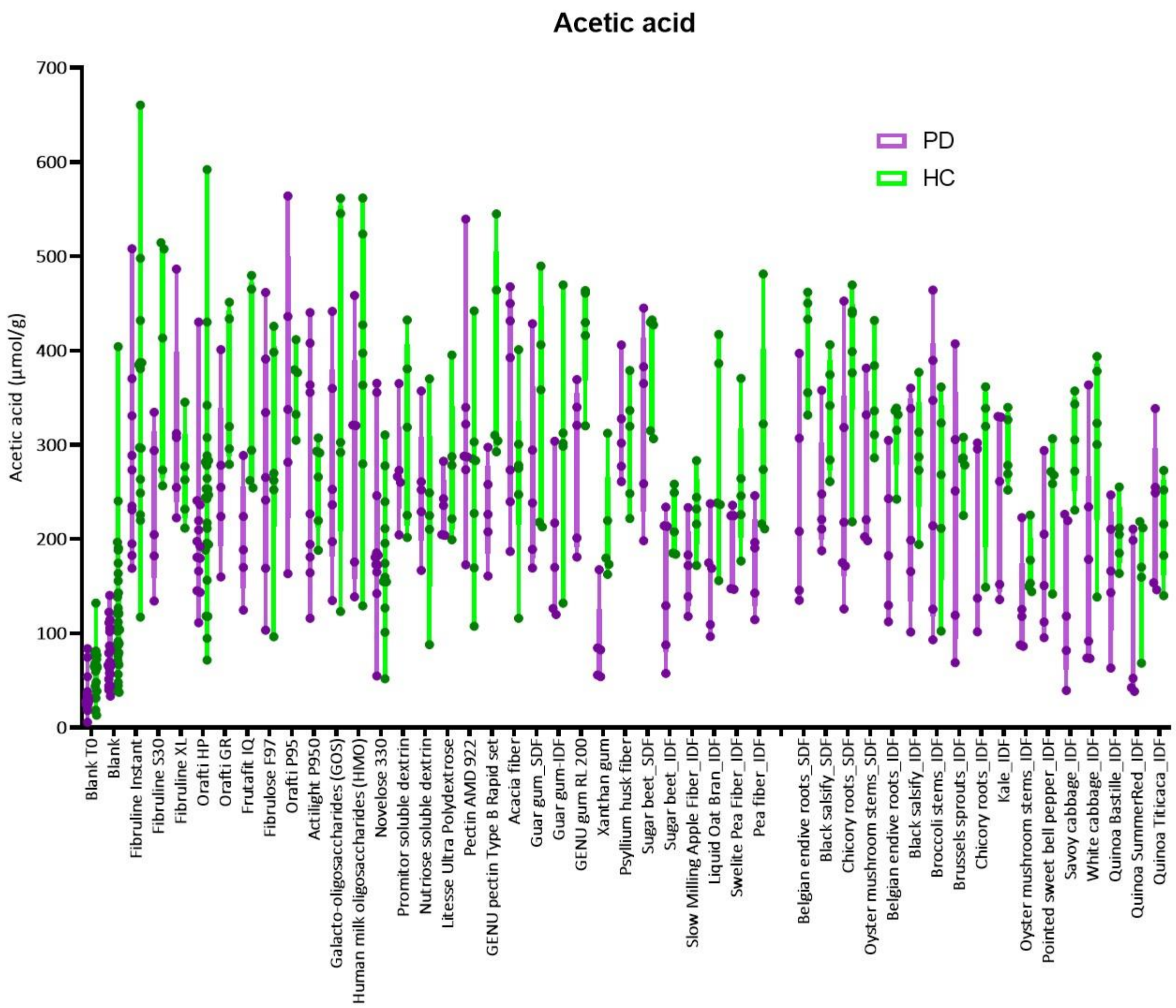

Results are shown as data points; PD, Parkinson's patients; HC, healthy controls; SDF, soluble dietary fiber; IDF, insoluble dietary fiber; Blank T0 is acetic acid concentration prior fermentation and Blank is acetic acid concentration after 24h of incubation without fiber

Supplementary Figure 2. Boxplot of propionic acid production post fermentation with fiber supplements, vegetable and quinoa fibers

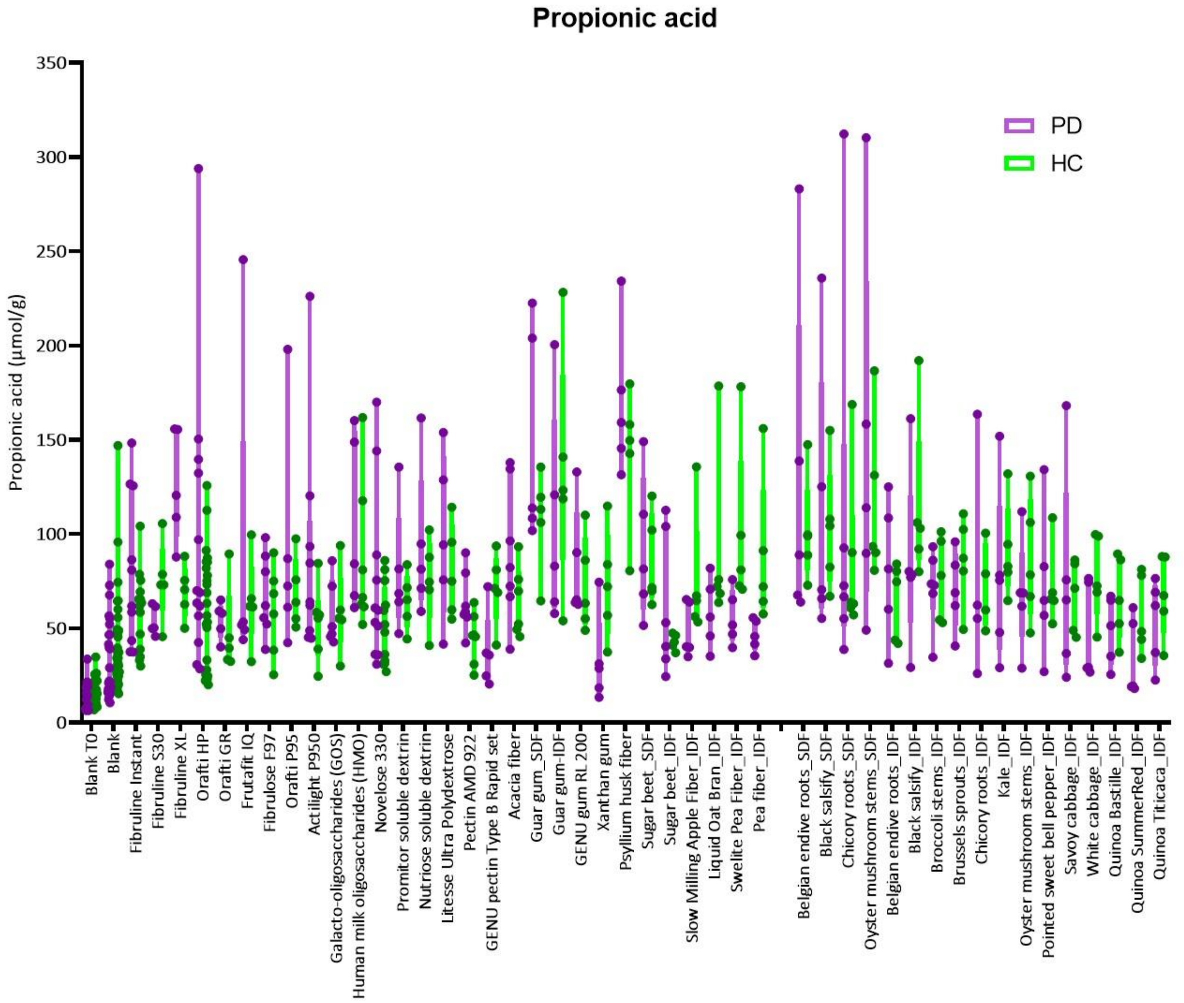

Results are shown as data points; PD, Parkinson's patients; HC, healthy controls; SDF, soluble dietary fiber; IDF, insoluble dietary fiber; Blank T0 is propionic acid concentration prior fermentation and Blank is propionic acid concentration after 24h of incubation without fiber

Supplementary Figure 3. Boxplot of butyric acid production post fermentation with fiber supplements, vegetable and quinoa fibers

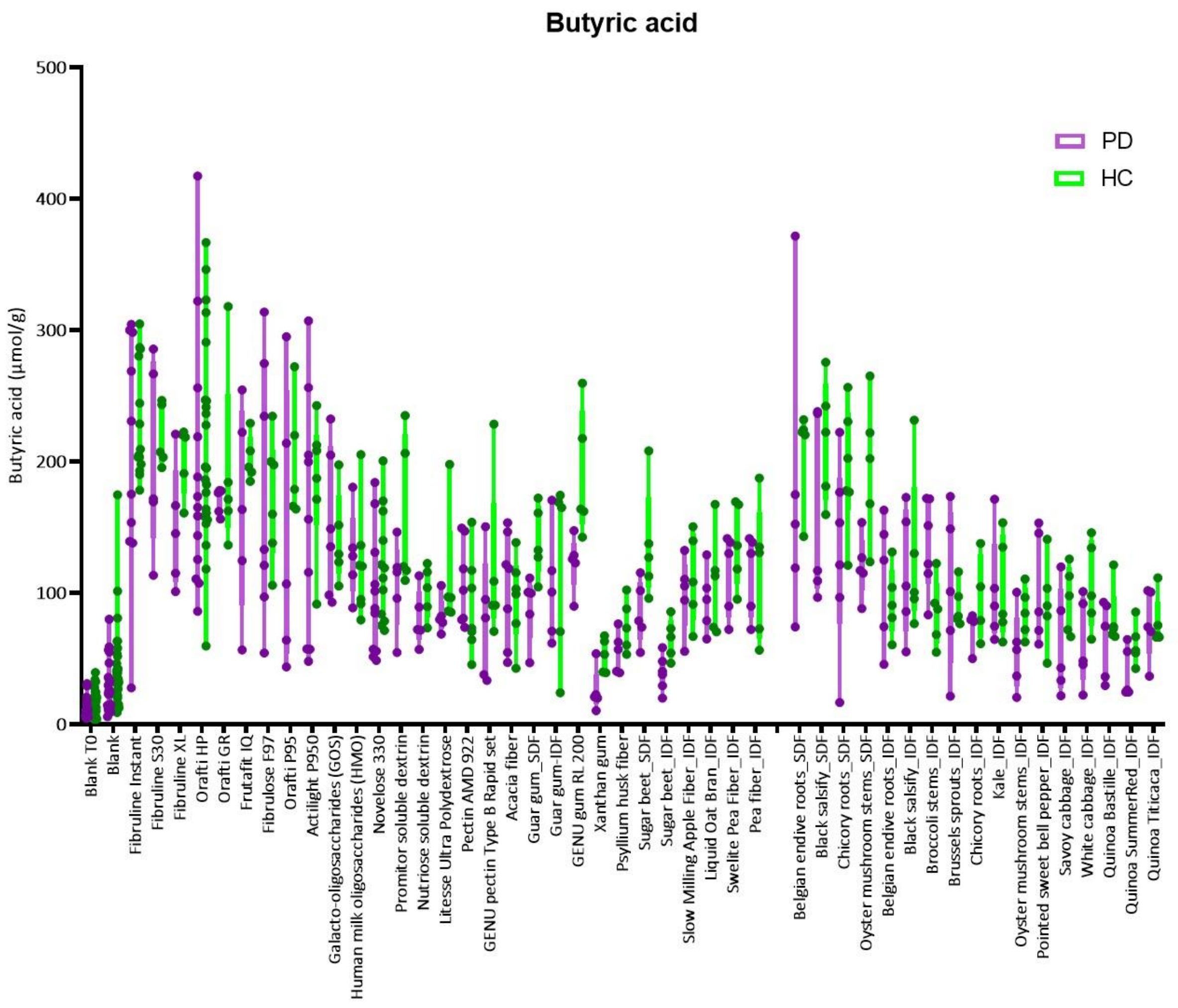

Results are shown as data points; PD, Parkinson's patients; HC, healthy controls; SDF, soluble dietary fiber; IDF, insoluble dietary fiber; Blank T0 is butyric acid concentration prior fermentation and Blank is butyric acid concentration after 24h of incubation without fiber

Supplementary Figure 4. Boxplot of isobutyric acid production post fermentation with fiber supplements, vegetable and quinoa fibers

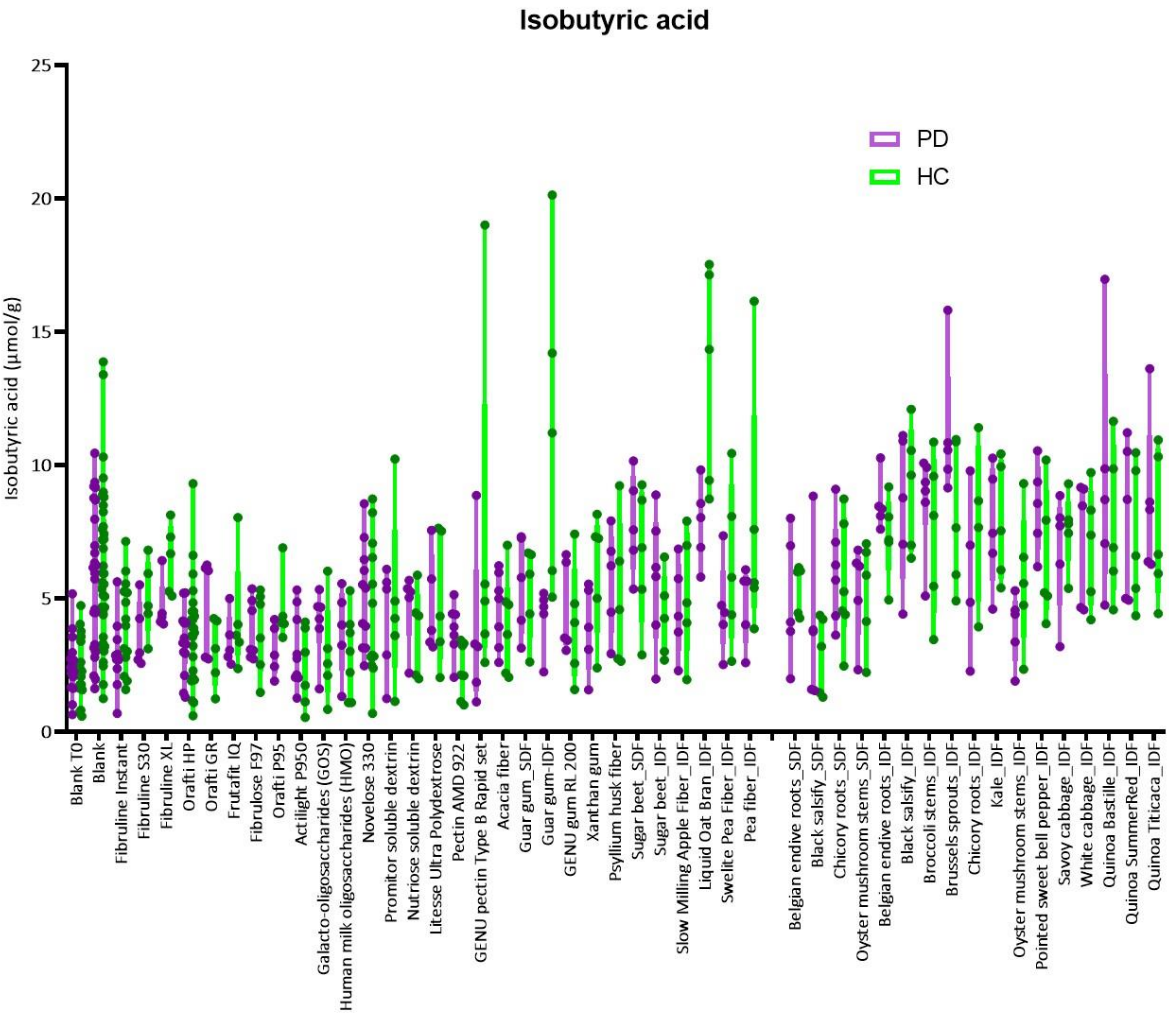

Results are shown as data points; PD, Parkinson's patients; HC, healthy controls; SDF, soluble dietary fiber; IDF, insoluble dietary fiber; Blank T0 is isobutyric acid concentration prior fermentation and Blank is isobutyric acid concentration after 24h of incubation without fiber

Supplementary Figure 5. Boxplot of valeric acid production post fermentation with fiber supplements, vegetable and quinoa fibers

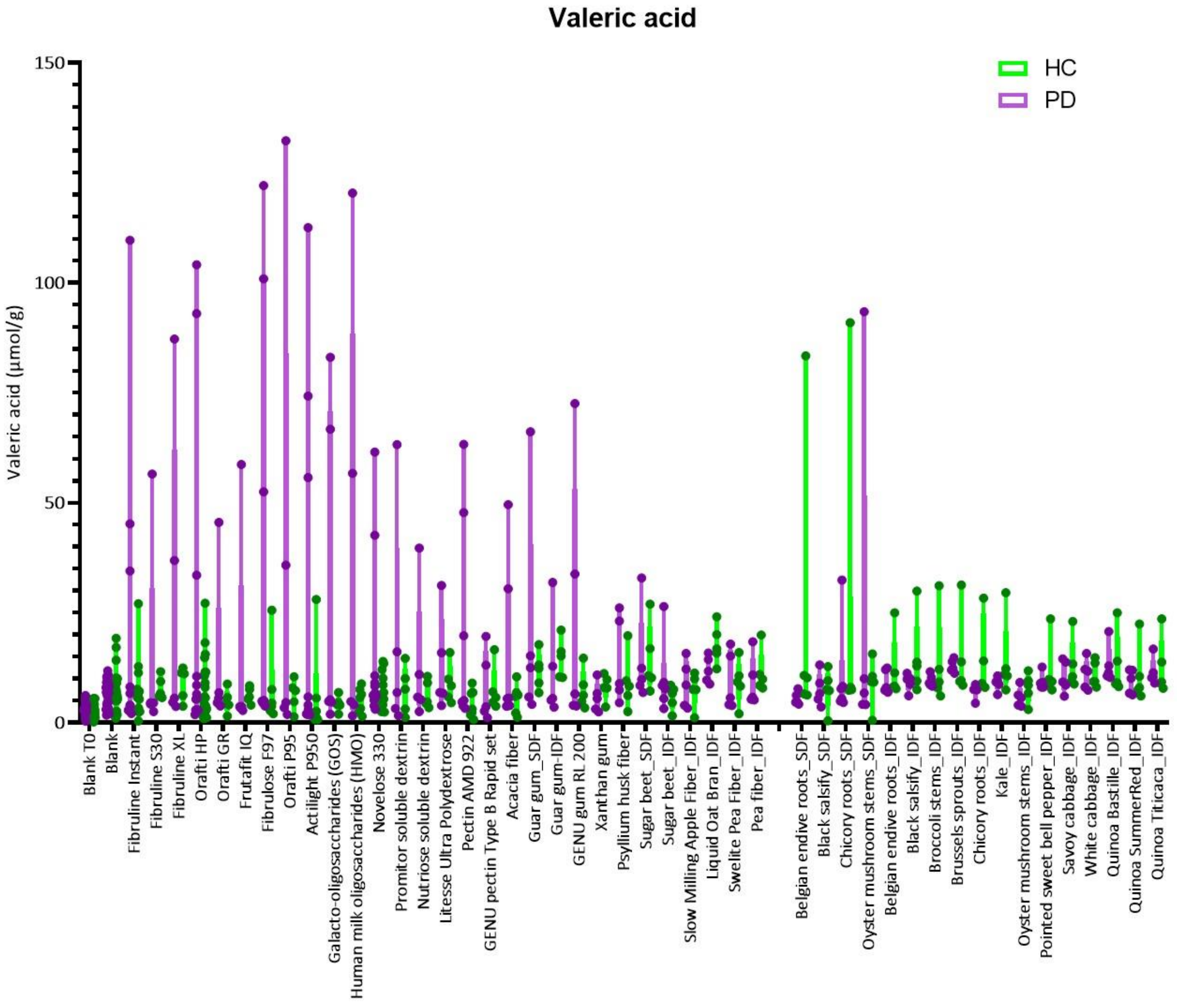

Results are shown as data points; PD, Parkinson's patients; HC, healthy controls; SDF, soluble dietary fiber; IDF, insoluble dietary fiber; Blank T0 is valeric acid concentration prior fermentation and Blank is valeric acid concentration after 24h of incubation without fiber

Supplementary Figure 6. Boxplot of isovaleric acid production post fermentation with fiber supplements, vegetable and quinoa fibers

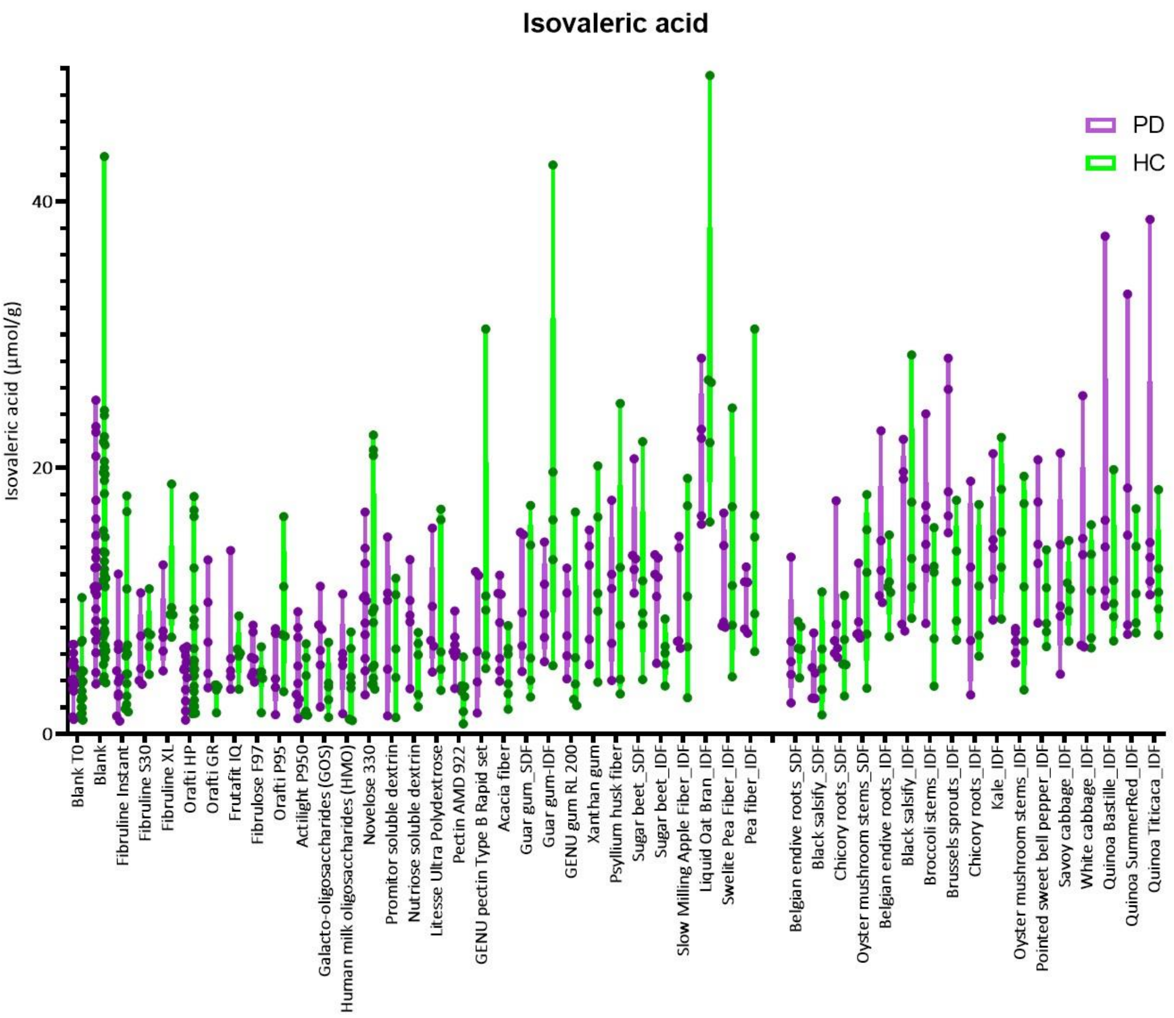

Results are shown as data points; PD, Parkinson's patients; HC, healthy controls; SDF, soluble dietary fiber; IDF, insoluble dietary fiber; Blank T0 is isovaleric acid concentration prior fermentation and Blank is isovaleric acid concentration after 24h of incubation without fiber

Supplementary Figure 7. Boxplot of total SCFA production post fermentation with fiber supplements, vegetable and quinoa fibers

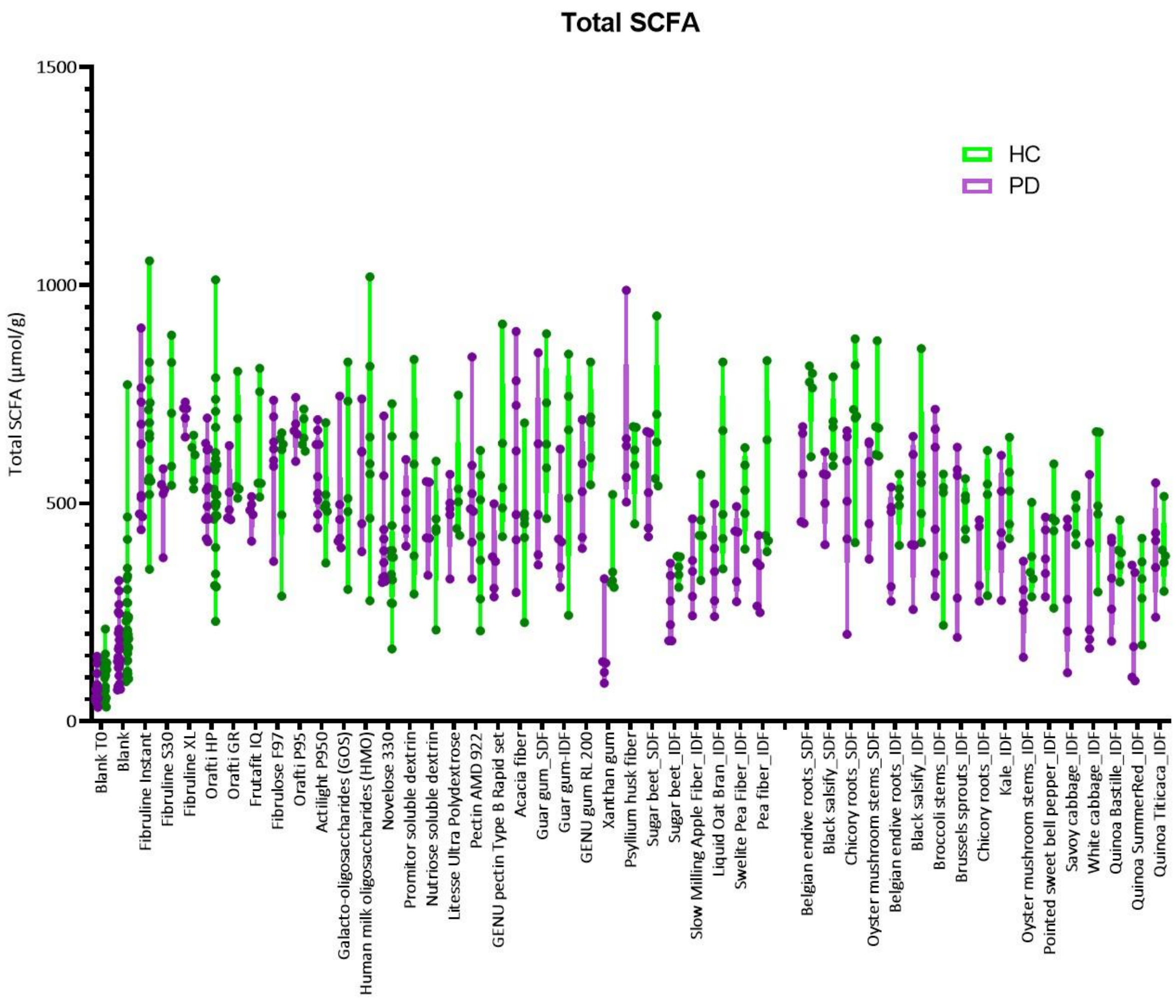

Results are shown as data points; PD, Parkinson's patients; HC, healthy controls; SDF, soluble dietary fiber; IDF, insoluble dietary fiber; Blank T0 is total SCFA concentration prior fermentation and Blank is total SCFA concentration after 24h of incubation without fiber

Supplementary Table 2. Overview of linear mixed model univariable analyses

Table 2 is available at https://doi.org/10.6084/m9.figshare.13238045.

Supplementary Table 3. Increase in butyrate production compared to blanks following fermentation of different fully fiber supplement types in Parkinson's Disease patients and healthy controls

Fiber types	Mean increase in butyrate production in PD (µmol/g)	Mean increase in butyrate production in HC (µmol/g)
Inulins	150,6±23,3	172,9±17,2
Oligosaccharides (FOS, GOS and HMO)	120,5±22,3	122,9±32,9
FOS	132,6±20,6	145,2±16,3
GOS	107,1±71,1	94,6±20,9
HMO	97,5±54,3	84,2±42,9
RS	68,5±35,0	87,3±40,4
RD	63,1±18,6	95,4±14,7
Polydextrose	63,4±5,8	100,7±53,1
Pectins	71,3±48,1	62,3±43,1
Hemicellulose, cellulose and lignin	56,1±30,2	73,6±34,3
Gums	59,2±41,4	75,9±51,5

Results are presented as mean ± SD; FOS, fructo-oligosaccharides; GOS, galacto-oligosaccharides; HMO, human milk oligosaccharides; RS, resistant starch; RD, resistant dextrin; PD, Parkinson's disease patients; HC, healthy controls.

Supplementary Figure 8. Increase in butyrate production compared to blanks following fermentation of different fully fiber supplement types in Parkinson's Disease patients and healthy controls

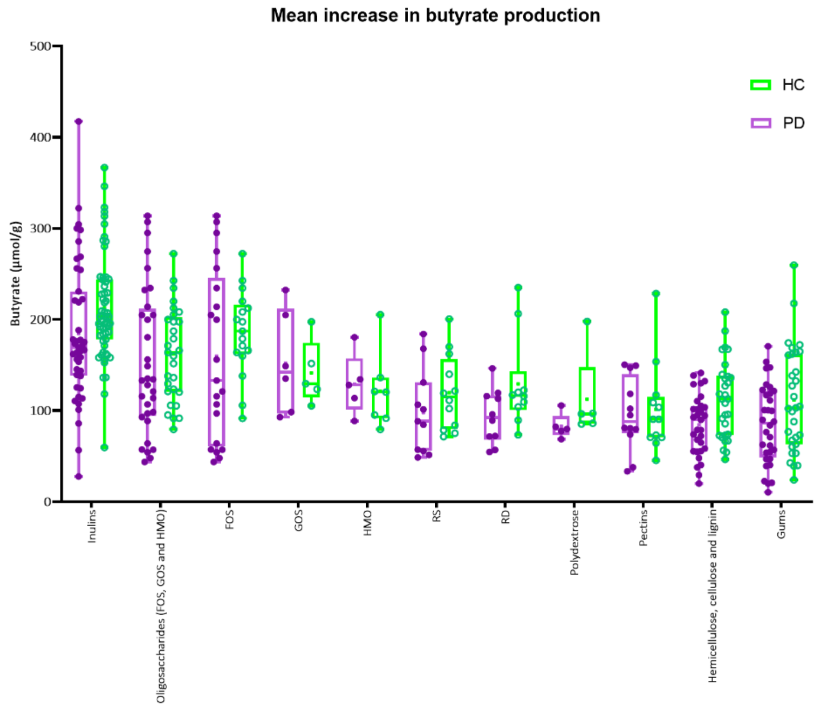

Butyrate production after fermentation with different fiber types. Results are shown as data points. FOS, fructo-oligosaccharides; GOS, galacto-oligosaccharides; HMO, human milk oligosaccharides; RS, resistant starch; RD, resistant dextrin; PD, Parkinson's disease patients; HC, healthy controls.

Supplementary Table 4. Kinetics parameters per fiber in Parkinson’s disease patients and healthy volunteers

	PD (n=3)				HC (n=3)			
	Blank	Inulin	FOS	RS	Blank	Inulin	FOS	RS
**AUC ((µmol/g)*h)**	1087 ± 512	8234 ± 2244	5167 ± 1085	3754 ± 602	1229 ± 269	9439 ± 670	8340 ± 712	5407 ± 471
**C_{max} (µmol/g)**	31,2 ± 11,5	236,1 ± 95,4	131,1 ± 43,3	106,9 ± 16,6	36,3 ± 9,6	236,0 ± 19,9	198,7 ± 26,9	163,6 ± 14,8
**T_{max} (h)**	42 ± 6	36 ± 6	36 ± 6	42 ± 6	36 ± 6	42 ± 6	20 ± 14	42 ± 6

Results are presented as mean ± SEM; all data are adjusted for baseline measurement; PD, Parkinson's disease patients; HC, healthy controls; SEM, standard error of the mean; AUC, area under the curve; C_{max}, maximum concentration; T_{max}, timepoint at which maximum concentration was produced.

Supplementary Table 5 pH changes during kinetics experiments per fiber in Parkinson’s disease patients and healthy volunteers

	PD (n=3)				HC (n=3)			
	Blank	Inulin	FOS	RS	Blank	Inulin	FOS	RS
Baseline	6,8 ± 0,3	6,8 ± 0,3	6,8 ± 0,3	6,8 ± 0,3	7,0 ± 0	7,0 ± 0	7,0 ± 0	7,0 ± 0
3h	6,8 ± 0,3	5,7 ± 1,2	5,7 ± 1,2	6,3 ± 0,8	7,0 ± 0	5,2 ± 0,8	5,2 ± 0,8	6,0 ± 0,5
6h	6,8 ± 0,3	5,0 ± 0,5	4,7 ± 0,3	5,7 ± 0,8	7,0 ± 0	5,0 ± 0,5	5,0 ± 0,5	5,5 ± 0
9h	6,8 ± 0,3	4,8 ± 0,3	4,5 ± 0	5,5 ± 0,5	7,0 ± 0	5,0 ± 0,5	4,8 ± 0,3	5,5 ± 0
12h	6,8 ± 0,3	4,8 ± 0,3	4,7 ± 0,3	5,5 ± 0,5	7,0 ± 0	5,0 ± 0,5	4,8 ± 0,3	5,5 ± 0
24h	6,8 ± 0,3	4,8 ± 0,3	4,7 ± 0,3	5,3 ± 0,3	7,0 ± 0	4,8 ± 0,3	4,8 ± 0,3	5,5 ± 0
30h	6,8 ± 0,3	4,8 ± 0,3	4,7 ± 0,3	5,3 ± 0,3	7,0 ± 0	4,8 ± 0,3	4,8 ± 0,3	5,5 ± 0
48h	6,8 ± 0,3	4,7 ± 0,3	4,7 ± 0,3	5,3 ± 0,3	7,0 ± 0	4,8 ± 0,3	4,8 ± 0,3	5,5 ± 0

Results are presented as mean ± SD; pH was measured using pH-indicator strips (4.5 – 10); PD, Parkinson’s patients; HC, healthy controls.

Supplementary Figure 9. Quantification results of bacteria belonging to *Clostridium Leptum* and *Clostridium Coccoides* groups in samples of Parkinson's disease patients and healthy volunteers

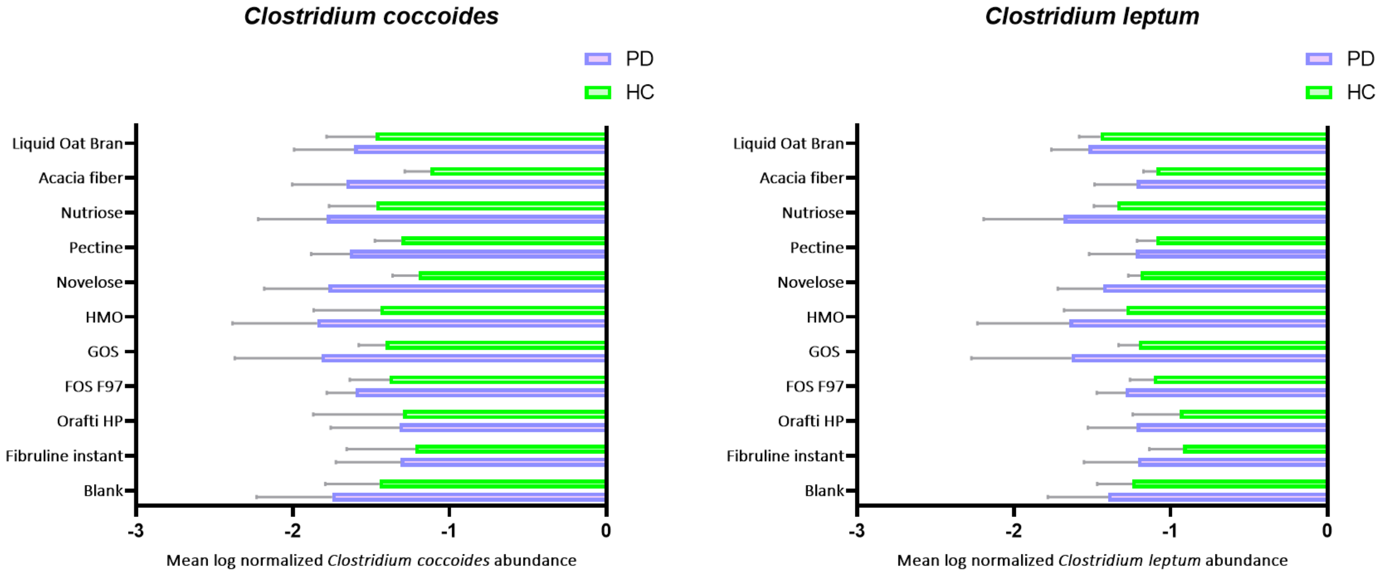

Mean butyrate-producing bacterial abundances in Parkinson's disease patients and healthy controls. Results are presented as mean ± SD; PD, Parkinson's disease patients; HC, healthy controls; Blanks are post-incubation blanks; Fibruline instant and Orafti HP are inulins; FOS F97 are fructo-oligosaccharides; GOS is galacto-oligosaccharides; HMO is human milk oligosaccharides; Novelose is resistant starch; pectine is pectin; nutriose is resistant dextrin; acacia fiber is a gum and liquid oat bran consists of a combination of hemicelluloses, cellulose and lignin. Potential effects of factors on butyrate-producers was analyzed using linear mixed models, sex and interaction effect of fiber type and PD diagnosis were added as fixed factors, participant was added as a random factor. Fiber type ($p<0.001$) and PD diagnosis ($p=0.02$ for C. leptum and $p=0.04$ for C. coccoides), independent from each other, significantly influenced C. leptum and coccoides abundances.

Supplementary Table 6. Increase in butyrate production compared to blanks following fermentation of different soluble vegetable fibers

Fiber types	Mean increase in butyrate production in PD (µmol/g)	Mean increase in butyrate production in HC (µmol/g)
Belgian endive roots (SDF)	133,8±107,3	172,0±27,1
Black salsify (SDF)	135,8±67,8	179,9±45,9
Chicory roots (SDF)	101,9±63,6	157,8±48,2
Oyster mushroom stems (SDF)	102,7±24,9	59,8±45,6

Results are presented as mean ± SD; SDF, soluble dietary fiber; PD, Parkinson's disease patients; HC, healthy controls

Supplementary Figure 10. Increase in butyrate production compared to blanks following fermentation of different soluble vegetable fibers

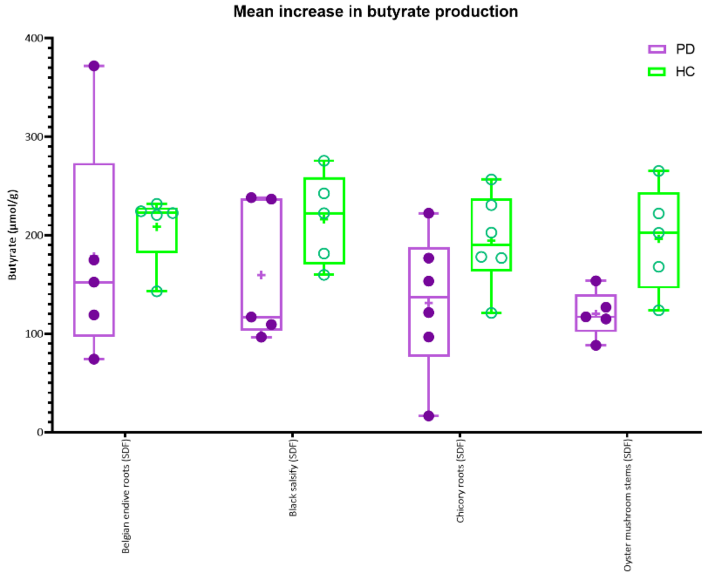

Mean butyrate concentration after fermentation with different fiber types. Results are shown as data points. PD, Parkinson's disease patients; HC, healthy controls; SDF, soluble dietary fiber; IDF, insoluble dietary fiber.

Supplementary Table 7. Overview of used dietary fiber substrates and their sources

Fiber substrate	Source
Oligosaccharides	
Fibrulose F97 *	Cosucra Groupe, Warcoing S.A., Belgium
Actilight 950P **	Caldic B.V., Belgium
Orafti P95	BENEO-Orafti S.A., Belgium
Biotis™ 2'-FL HMO*	FrieslandCampina Ingredients, The Netherlands
GOS *	DuPont Nutrition & Biosciences, Denmark
Inulins	
Orafti HP */**	BENEO-Orafti S.A., Belgium
Orafti GR	BENEO-Orafti S.A., Belgium
Fibruline Instant *	Cosucra Groupe, Warcoing S.A., Belgium
Fibruline XL	Cosucra Groupe, Warcoing S.A., Belgium
Fibruline S30	Ingrizo Food Intelligence NV, Belgium
Frutafit IQ	Sensus, The Netherlands
Pectins	
Grindsted Pectin AMD 922 *	DuPont Nutrition & Biosciences, Denmark
Genu Pectin type B	CP Kelco, United States of America (USA)
Gums	
Genu gum RL 200 locust bean gum	CP Kelco, United States of America (USA)
Acacia fiber *	Caldic B.V., Belgium
Xanthan gum	Snick Euroingredients NV, Belgium
Guar gum	Snick Euroingredients NV, Belgium
Psyllium	Solina Group SAS, United Kingdom (UK)
Resistent dextrin/starch	
Novelose 330 */**	Caldic B.V., Belgium
Promitor	Tate &Lyle, United Kingdom (UK)
Nutriose FM06 *	Barentz BV, The Netherlands
Polydextrose	
Litesse Ultra ™ IP Powder	DuPont Nutrition & Biosciences, Denmark
Rest group (hemicelluloses, cellulose, lignin)	
Slow Milling Apple fiber	Snick Euroingredients NV, Belgium
Liquid Oat Bran 10 Instant *	Ingrizo Food Intelligence NV, Belgium
Peafiber I 50M	Barentz BV, The Netherlands
Swelite - Peafiber	Snick Euroingredients NV, Belgium
Sugar beet fiber	Snick Euroingredients NV, Belgium
Vegetable and quinoa substrates	
Belgian endive roots	Inagro, Belgium
Black Salsify	Greenyard Prepared Belgium NV, Belgium
Broccoli stems	Greenyard Prepared Belgium NV, Belgium
Brussels sprouts	Local farmer
Cabbage	Greenyard Prepared Belgium NV, Belgium
Chicory roots	Local farmer
Kale	Greenyard Prepared Belgium NV, Belgium
Oyster mushroom stems	Beefygreen, the Netherlands
Pointed sweet bell pepper	Barver bvba, Belgium
Quinoa Bastille	ILVO, Unit plant sciences, Belgium
Quinoa Summer Red	ILVO, Unit plant sciences, Belgium
Quinoa Titicaca	ILVO, Unit plant sciences, Belgium
White cabbage	Greenyard Prepared Belgium NV, Belgium

**, fiber substrates were used in qPCR analyses of the Clostridium clusters; **, fiber substrates were used to assess SCFA production kinetics; HMO, human milk oligosaccharides; 2'-FL, 2'fucosyllactose*

Supplementary Table 8. Composition and anti-oxidative properties of vegetable and quinoa original powders and fibers

	Original powder: Proteins (% DWB)	Original powder: Non-RS (% DWB)	Original powder: RS (% DWB)	Original powder: Polyphenols$_{TOT}$ (µg/kg DWB)	Original powder: DPPH (TE µmol/g DWB)	Soluble dietary fiber fraction: Mono-& disaccharides (mg/g)	Soluble dietary fiber fraction: SDF (mg/g)	Soluble dietary fiber fraction: Proteins (% DWB)	Soluble dietary fiber fraction: Polyphenols$_{TOT}$ (µg/kg DWB)	Soluble dietary fiber fraction: ORAC (TE µmol/g DWB)	Soluble dietary fiber fraction: DPPH (TE µmol/g DWB)	Insoluble dietary fiber fraction: Mono-& disaccharides (mg/g)	Insoluble dietary fiber fraction: SDF (mg/g)	Insoluble dietary fiber fraction: Proteins (% DWB)	Insoluble dietary fiber fraction: Non-RS (% DWB)	Insoluble dietary fiber fraction: RS (% DWB)	Insoluble dietary fiber fraction: Polyphenols$_{TOT}$ (µg/kg DWB)	Insoluble dietary fiber fraction: ORAC (TE µmol/g DWB)	Insoluble dietary fiber fraction: DPPH (TE µmol/g DWB)
BER	8,15	1,48 ± 0,25	0,07 ± 0,006	62117,9 ± 2261,1	25,2 ± 0,4	235,6 ± 6,1	553,6 ± 12,2	11,17	10319,7 ± 244,6	31,0 ± 2,5	<LOD	34,3 ± 0,5	40,5 ± 3,3	5,82	0,26 ± 0,05	0,09 ± 0,009	2908,4 ± 104,9	6,8 ± 5,6	1,1 ± 0,04
BS	20,75	11,94 ± 0,28	0,55 ± 0,009	2615,5 ± 99,1	11,0 ± 0,1							71,9 ± 0,4	55,6 ± 4,1	14,97	0,82 ± 0,05	0,13 ± 0,01	168,2 ± 9,4	6,7 ± 1,1	1,2 ± 0,01
BRS	25,76	9,22 ± 0,57	0,17 ± 0,007	3898,8 ± 229,3	18,3 ± 1,2							46,4 ± 0,2	29,4 ± 0,3	19,39	0,74 ± 0,01	0,08 ± 0,002	102,1 ± 2,4	12,5 ± 3,3	2,5 ± 0,02
CAB	19,2	10,59 0,41	0,26 ± 0,003	1591,2 ± 171,1	8,1 ± 0,2							46,2 ± 0,3	21,8 ± 0,3	15,18	0,72 ± 0,03	0,09 ± 0,01	1262,0 ± 41,9	12,1 ± 3,3	1,4 ± 0,1
CR	5,09	0,68 ± 0,12	0,02 ± 0,004	71115,9 ± 8785,4	14,5 ± 1,1	75,3 ± 6,4 **	870,6 ± 10,2	3,56	5664,3 ± 86,5	14,6 ± 0,8	<LOD	31,2 ± 0,1	81,5 ± 3,0	9,18	0,17 ± 0,02	0,05 ± 0,01	2307,8 ± 38,1	2,5 ± 1,7	0,6 ± 0,02
KA	24,16	6,46 ± 0,35	0,10 ± 0,01	18757,9 ± 646,9	38,7 ± 0,5							29,8 ± 0,05	17,6 ± 0,5	21,54	0,82 ± 0,31	0,06 ± 0,01	5277,7 ± 15,8	21,1 ± 7,1	2,1 ± 0,07
OMS	8,34	12,96 ± 0,05	0,24 ± 0,008	760,32 ± 246,7	14,4 ± 1,4	56,2 ± 1,8	560,7 ± 3,6	11,62	259,8 ± 9,9	18,3 ± 2,3	3,3 ± 0,2	35,5 ± 12,3	34,6 ± 1,5	4,13	1,10 ± / *	0,11 ± 0,003	36,8 ± 1,7	10,7 ± 1,8	0,6 ± 0,04
PSBP	10,64	19,03 ± 0,62	0,12 ± 0,004	33602,0 ± 1874,9	81,4 ± 3,0							61,4 ± 0,08	53,4 ± 0,4	12,65	1,46 ± 0,13	0,05 ± 0,01	3472,7 ± 42,1	11,6 ± 2,5	1,7 ± 0,02
BLS	17,53	0,81 ± 0,11	0,08 ± 0,01	25308,2 ± 1120,4	17,0 ± 1,0	214,3 ± 2,5	560,0 ± 0,09	16,51	221,0 ± 96,9	12,6 ± 4,0	1,2 ± 1,4	53,3 ± 0,02	67,7 ± 14,5	17,22	0,13 ± 0,02	0,07 ± 0,006	1926,2 ± 81,0	11,6±2,5	2,6 ± 0,02
WCAB	18,79	13,74 ± 0,18	0,38 ± 0,007	995,38 ± 38,8	20,2 ± 1,2							61,3 ± 0,3	40,3 ± 0,02	12,75	1,45 ± 0,02	0,18 ± 0,01	1959,4 ± 209,9	7,1 ± 2,2	0,8 ± 0,05
QBA	13,34	64,40 ± 1,65	0,07 ± 0,001	854,0 ± 31,9	2,2 ± 0,1							33,8 ± /	85,0 ± /	29,89	7,15 ± 0,01	0,31 ± 0,003	506,2 ± 37,9	18,1 ± 1,0	0,8 ± 0,01
QTI	13,64	56,41 ± 0,16	0,19 ± 0,003	4786,9 ± 248,4	5,8 ± 0,01							27,8 ± 0,05	38,4 ± 2,4	33,37	3,12 ± 0,16	0,25 ± 0,01	2738,5 ± 64,0	16,6 ± 0,3	0,9 ± 0,05
QSR	13,58	64,15 ± 0,42	0,08 ± 0,006	2801,5 ± 103,6	5,8 ± 0,1							11,6 ± 0,005	23,7 ± 5,7	23,77	5,05 ± 0,03	0,85 ± 0,03	1426,5 ± 137,2	16,1 ± 2,2	1,9 ± 0,03

*Results are presented as mean ± SD; **pectin wasn't included in total soluble dietary fiber because of uncertainties of quantification; Results of DPPH are an estimation of the Trolox Equivalents at an inhibition of 50%. For samples with no IC50, TE result is based upon the measurement of the highest concentration; *replicate got lost during analysis; DWB, dry weight based; TE, Trolox equivalent; <LOD, below limit of detection; RS, resistant starch, Polyphenols$_{TOT}$, total polyphenols; BER, Belgian endive roots; BS, broccoli stems; BRS, Brussels sprouts; CAB, cabbage; CR, chicory roots; KA, kale; OMS, oyster mushroom stems; PSBP, pointed sweet bell pepper; BLS, black salsify; WCAB, white cabbage; QBA, quinoa Bastille; QTI, quinoa Titicaca; QSR, quinoa Summer red; SDF, soluble dietary fiber*

Supplementary Table 9: Soluble carbohydrate composition of vegetable and quinoa fibers

	Inulin (mg/g)	Pectin (mg/g)	Oligosaccharides (mg/g)	Cellobiose (mg/g)	Unknown soluble dietary fiber (mg/g)	Sucrose (mg/g)	Glucose (mg/g)	Fructose (mg/g)	Unknown mono- and disaccharides (mg/g)
BER - IDF	<LOD	22,1 ± 0,3	4,3 ± 0,2	<LOD	14,1 ± 3,4	6,8 ± 0,2	0,4 ± 0,2	6,3 ± 0,08	20,8 ± 0,08
BER - SDF	300,8 ± 6,6	<LOD	237,3 ± 5,7	<LOD	15,4 ± 0,009	152,1 ± 5,7	<LOD	83,5 ± 0,4	<LOD
BLS - IDF	<LOD	21,3 ± 0,4	4,4 ± 2,4	<LOD	42,0 ± 12,4	5,1 ± 0,1	0,6 ± 0,03	2,8 ± 0,04	44,8 ± 2,5
BLS - SDF	321,8 ± 6,2	<LOD	221,6 ± 5,7	<LOD	16,7 ± 0,4	16,2 ± 0,09	11,7 ± 1,0	81,1 ± 1,6	105,4 ± 0,2
BS - IDF	<LOD	9,8 ± 4,1	1,0 ± 0,2	1,02 ± 0,02	4,4 ± 0,1	9,5 ± 0,04	5,8 ± 0,1	9,4 ± 0,2	44,4 ± 0,06
BRS - IDF	<LOD	9,2 ± 0,4	2,3 ± 0,6	<LOD	19,6 ± 0,5	16,4 ± 0,3	5,8 ± 0,2	2,7 ± 0,01	21,5 ± 0,7
CAB - IDF	<LOD	1,2 ± 0,3	2,1 ± 0,03	<LOD	20,3 ± 0,06	15,0 ± 0,2	6,3 ± 0,05	4,7 ± 0,2	20,2 ± 0,006
CR - IDF	NA***	28,9 ± 2,8	6,2 ± 0,5	<LOD	46,3 ± 0,3	4,8 ± 0,2	0,2 ± 0,35	1,9 ± 0,2	24,3 ± 0,7
CR - SDF	634,1 ± 3,2	NA**	167,0 ± 5,8	<LOD	69,5 ± 1,2	56,5 ± 4,9	<LOD	18,7 ± 1,7	<LOD
KA - IDF	<LOD	0,18 ± 0,3	0,7 ± 0,1	<LOD	16,8 ± 0,8	3,1 ± 0,01	2,5 ± 0,09	2,8 ± 0,04	21,4 ± 0,2
OMS - IDF	<LOD	<LOD	4,9 ± 0,6	12,3 ± 0,07	18,8 ± 1,4	<LOD	13,3 ± 11,9	<LOD	22,2 ± 0,3
OMS - SDF	<LOD	<LOD	11,8 ± 5,3	386,3 ± 1,3	162,7 ± 3,0	<LOD	40,2 ± 1,2	<LOD	16,0 ± 0,5
PSBS - IDF	<LOD	32,2 ± 1,4	2,3 ± 0,3	<LOD	20,7 ± 0,7	5,6 ± 0,08	13,0 ± 0,1	19,2 ± 0,02	23,7 ± 0,07
QBA - IDF*	0 ± /	0 ± /	67,5 ± /	16,9 ± /	10,0 ± /	2,0 ± /	29,4 ± /	<LOD	2,5 ± /
QSR - IDF	<LOD	1,0 ± 1,4	16,8 ± 0,7	3,05 ± 4,2	0,4 ± 0,04	1,2 ± 0,05	8,2 ± 0,05	<LOD	2,2 ± 0,01
QTI - IDF	<LOD	2,5 ± 3,5	28,7 ± 2,4	9,4 ± 0,1	7,5 ± 0,3	1,3 ± 0,03	25,0 ± 0,001	<LOD	1,5 ± 0,02
WCAB - IDF	<LOD	13,2 ± 0,2	3,4 ± 0,07	<LOD	23,7 ± 0,1	21,1 ± 0,04	8,3 ± 0,2	6,0 ± 0,1	25,9 ± 0,01

*Results are presented as mean ± SD; IDF, insoluble dietary fiber; SDF, soluble dietary fiber; * replicate got lost during analysis; NA, not applicable; ** there was difficulty with integration and thus quantification of pectin in the samples of chicory root – SDF; *** inulin was found in the sample, however peaks could not be separated from unkown dietary fiber; BER, Belgian endive roots; BS, broccoli stems; BRS, Brussels sprouts; CAB, cabbage; CR, chicory roots; KA, kale; OMS, oyster mushroom stems; PSBP, pointed sweet bell pepper; BLS, black salsify; WCAB, white cabbage; QBA, quinoa Bastille; QTI, quinoa Titicaca; QSR, quinoa Summer red*

Supplementary Table 10. Mono- and disaccharide composition of fiber supplements

Sample	Mono- and disaccharides (mg/g)
Orafti HP - SDF	3,1 ± 0,001
Fibruline Instant - SDF	54,6 ± 1,7
Fibruline XL - SDF	27,3 ± 1,9
Fibruline S30 - SDF	53,1 ± 0,2
Orafti GR - SDF	65,5 ± 1,0
Frutafit IQ - SDF	68,8 ± 5,3
Fibrulose F97 - SDF	55,6 ± 0,3
Orafti P95 - SDF	73,4 ± 0,06
Actilight 950P - SDF	40,4 ± 1,8
GOS - SDF	220,0 ± 0,5
Human milk oligosaccharide - SDF	24,4 ± 2,0
Novelose 330 - IDF	114,2 ± 0,1
Promitor - SDF	129,8 ± 0,9
Nutriose FM06 - SDF	10,3 ± 0,1
Litesse Ultra ™ IP Powder - SDF	94,3 ± 2,5
Acacia fiber - SDF	15,9 ± 0,3
Psyllium - SDF	2,7 ± 0,04
Genu gum RL 200 - SDF	24,7 ± 0,7
Xanthan gum - SDF	<LOD
Guar gum - SDF	1,4 ± 0,06
Guar gum - IDF	61,9 ± 0,1
Grindsted Pectin AMD 922 - SDF	34,5 ± 0,1
Genu Pectin type B - SDF	1,4 ± 0,0004
Liquid Oat Bran - IDF	11,8 ± 0,3
Apple fiber - IDF	34,5 ± 0,1
Peafiber I 50M - IDF	47,5 ± 0,2
Swelite - Peafiber - IDF	60,4 ± 0,09
Sugar beet fiber - SDF	34,6 ± 0,7
Sugar beet fiber – IDF*	3,8 ± /

*Results are presented as mean ± standard deviation; IDF, insoluble dietary fiber; SDF, soluble dietary fiber; *, replicate got lost during analysis*

Supplementary Table 11. Overview of qPCR parameters: primer sequences, primer concentration, amplification and melting curve analysis

Target	Primer	Concentration (nM)	Sequence (5'-3')	Amplicon length	Reference
Clostridium Leptum group	sg-Clept-F	250	GCACAAGCAGTGGAGT	246	[5]
	sg-Clept-R	250	CTTCCTCCGTTTTGTCAA		
Clostridium Coccoides group	g-Cocc-F	250	AAATGACGGTACCTGACTAA	440	[6]
	g-Cocc-R	250	CTTTGAGTTTCATTCTTGCGAA		
16S rRNA Bacteria	BAC338-F	100	ACTCTACGGGAGGCAG	473	[7, 8]
	BAC508-R	100	ATTACCGCGGCTGCTGG		
